# Enhancing Oxygen Evolution Electrocatalysis in Heazlewoodite: Unveiling the Critical Role of Entropy Levels and Surface Reconstruction

**DOI:** 10.1002/adma.202501186

**Published:** 2025-04-07

**Authors:** Hangning Liu, Xinghang Liu, Anbang Sun, Cuijuan Xuan, Yingjun Ma, Zixuan Zhang, Hui Li, Zexing Wu, Tianyi Ma, Jie Wang

**Affiliations:** ^1^ Qingdao Engineering Research Center of Agricultural Recycling Economy Materials College of Chemistry and Pharmaceutical Sciences Qingdao Agricultural University Qingdao 266109 P. R. China; ^2^ School of Industrial and Information Engineering Politecnico di Milano Milano 20133 P. R. China; ^3^ Shandong Institute of Non‐Metallic Materials Jinan 250031 China; ^4^ Centre for Atomaterials and Nanomanufacturing (CAN) RMIT University Melbourne VIC 3000 Australia; ^5^ State Key Laboratory Base of Eco‐chemical Engineering College of Chemistry and Molecular Engineering Qingdao University of Science & Technology 53 Zhengzhou Road Qingdao 266042 P. R. China

**Keywords:** electrocatalysis, entropy engineering, nickel sulfide, oxygen evolution reaction, surface reconstruction

## Abstract

Entropy engineering has proven effective in enhancing catalyst electrochemical properties, particularly for the oxygen evolution reaction (OER). Challenges persist, however, in modulating entropy and understanding the dynamic reconfiguration of high‐entropy sulfides during OER. In this study, an innovative in situ corrosion method is introduced to convert low‐valent nickel on a nickel foam substrate into high‐entropy heazlewoodite (HES/NF), significantly boosting OER performance. By synthesizing a series of low‐, medium‐, and high‐entropy heazlewoodites, the intrinsic factors influence catalyst surface evolution and electrocatalytic activity is systematically explored. Employing a combination of in situ and ex situ characterization techniques, it is observed that HES/NF dynamically transforms into a stable hydroxide oxide (MOOH)‐sulfide composite under OER conditions. This transition, coupled with lattice distortion, optimizes the electrostatic potential distribution, ensuring superior catalytic activity and preventing surface sulfide deactivation through the formation of stable HES‐MOOH species. This synergy enables HES/NF to achieve remarkably low overpotentials: 172.0 mV at 100.0 mA cm^−2^ and 229.0 mV at an extreme current density of 300.0 mA cm^−2^. When paired with a Pt/C cathode, HES/NF exhibits rapid kinetics, outstanding stability, and exceptional water‐splitting performance. The scalable, cost‐effective approach paves the way for advanced electrocatalyst design, promising breakthroughs in energy storage and conversion technologies.

## Graphical abstract

An innovative high‐entropy heazlewoodite is synthesized, where the formation of a high‐entropy phase significantly enhanced oxygen evolution reaction activity, and the oxidation potential evolved stabilized high‐entropy sulfide‐hydroxide oxide species at the interface improved electrochemical stability.

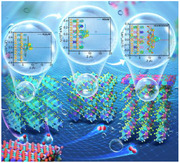

## 1 Introduction

Hydrogen energy is increasingly acknowledged as a pivotal element in advancing the transition to a sustainable, low‐carbon future.^[^ ^1^, ^2^ ^]^ As an energy carrier,^[^ ^3^ ^]^ it holds substantial promise for addressing both global energy shortages and the urgent need to mitigate carbon emissions.^[^ ^4^ ^]^ Traditional industrial methods for hydrogen production, such as steam methane reforming, are energy‐intensive and release significant amounts of CO₂,^[^ ^5^, ^6^ ^]^ contributing to environmental issues. In contrast, electrochemical water splitting offers a cleaner and more sustainable approach by using electricity—ideally sourced from renewable energy—to generate hydrogen and oxygen, producing zero carbon emissions and presenting an efficient pathway for green hydrogen production.^[^ ^7^ ^]^ A critical step in hydrogen production via water electrolysis is the oxygen evolution reaction (OER), a half‐reaction involving the transfer of four electrons and protons.^[^ ^8^, ^9^ ^]^ However, the OER faces is hindered by inherently sluggish reaction kinetics,^[^ ^10^, ^11^ ^]^ necessitating a high overpotential to overcome its substantial energy barrier.^[^ ^12^, ^13^ ^]^ This requirement not only increases energy consumption but also significantly reduces the overall efficiency of water‐splitting technologies.^[^ ^14^, ^15^ ^]^ Consequently, the sluggish reaction kinetics of the 4‐electron OER represents a major bottleneck in the practical implementation of electrochemical hydrogen production.^[^ ^16^, ^17^ ^]^ To date, noble metal catalysts, such as ruthenium‐ (Ru)^[^ ^18^ ^]^ and iridium‐ (Ir) based catalysts,^[^ ^19^, ^20^ ^]^ have demonstrated outstanding OER activity.^[^ ^21^, ^22^ ^]^ However, their large‐scale application faces critical challenges due to their scarcity, high cost, and limited long‐term durability.^[^ ^23^, ^24^ ^]^ This underscores an urgent need to develop alternative catalysts that are abundant cost‐effective, and derived from Earth's readily available resources.^[^ ^25^, ^26^, ^27^ ^]^

Transition metal‐based catalysts have emerged as highly promising candidates for the OER, owing to their favorable electrochemical properties, cost‐effectiveness, and relative abundance. Among these, transition metal sulfides have garnered significant attention due to their high density of reactive sites, high conductivity, and tunable electron structure. Moreover, transition metal sulfides are likely to undergo phase transformations at electrochemical interfaces during the OER, driven by the high oxidation potential. These transformations result in the formation of active oxides or hydroxides, which typically exhibit superior catalytic properties.^[^ ^16^, ^17^ ^]^ Such interfacial reconfiguration can be further influenced by doping or partial substitution, which modifies local electronic structures and valence states. One notable example is nickel trisulfide (Ni_3_S_2_),^[^ ^28^ ^]^ also named as heazlewoodite, which undergoes surface transformation during the OER, leading to the formation of nickel oxyhydroxide (NiOOH) species widely regarded as the true active sites for electrocatalysis. During this surface reconstruction process, nickel atoms transition to higher oxidation states, such as NiOOH. These transformations have been shown to enhance catalytic activities. Nevertheless, a comprehensive understanding of the complex interplay between structural evolution and catalytic performance for the OER, remains an area of active research and considerable interest.^[^ ^29^ ^]^

High‐entropy materials have recently emerged as a promising class of electrocatalysts, distinguished by their unique chemical environments arising from the incorporation of multiple principal elements and high configurational entropy.^[^ ^30^, ^31^ ^]^ This inherent heterogeneity enables adjustable electronic structures and provides a high density of active sites, making high‐entropy compounds particularly attractive for catalytic applications.^[^ ^32^, ^33^, ^34^ ^]^ Although high‐entropy sulfides have demonstrated potential, their application in the OER is still in its early stages.^[^ ^35^, ^36^, ^37^ ^]^ The effects of entropy engineering on catalyst morphology and catalytic activity remain poor understood and insights into dynamic interface evolution of these catalysts during reactions are limited.^[^ ^38^, ^39^ ^]^ Unravelling the relationship between entropy engineering and catalytic properties, as well as elucidating the mechanisms of surface transformations during catalysis, could pave the way for reducing energy barriers and enhancing reaction kinetics. Such advancements hold significant promises for improving the practical application of high‐entropy catalysts in sustainable hydrogen production.

Herein, we present an innovative in situ corrosion strategy for the synthesis of a series of heazlewoodite catalysts with varying levels of entropy. Systematically exploration was employed to study the increases of entropy in regulating the surface evolution of heazlewoodites and the electrocatalytic activity toward OER. By comparing the heazlewoodite catalysts with increasing levels of entropy on nickel foam (NF) substrates, the fundamental principles governing these transformations and their impact on catalytic performance is elucidated. Utilizing a comprehensive suite of in situ (in situ EIS) and ex situ (ex situ SEM, ex situ Raman and ex situ TEM) characterization techniques, high entropy sulfide on the NF substrate (HES/NF) dynamically transforms into a stable HES‐MOOH interface during the continuous OER process. This transition is accompanied by lattice distortion, which not only optimizes the electrostatic potential distribution but also enhances the stability of the catalyst while maintaining superior OER activity. Density functional theory (DFT) calculations were performed to study the properties of the adsorption strength toward oxygen‐containing intermediates from Ni_3_S_2_/NF to HES/NF before and after surface reconstruction. Additionally, these calculations explored how charge redistribution affects electrocatalytic stability while maintaining OER activity. It is speculated that the surface HES‐MOOH evolution is responsible for providing excellent OER activities, stability and water splitting performance when coupled with Pt/C cathode. This work aims to provide deeper insights into the reaction mechanisms and establish the groundwork for future high‐performance electrocatalyst designs.

## 2 Results and Discussion

**Figure 1:**
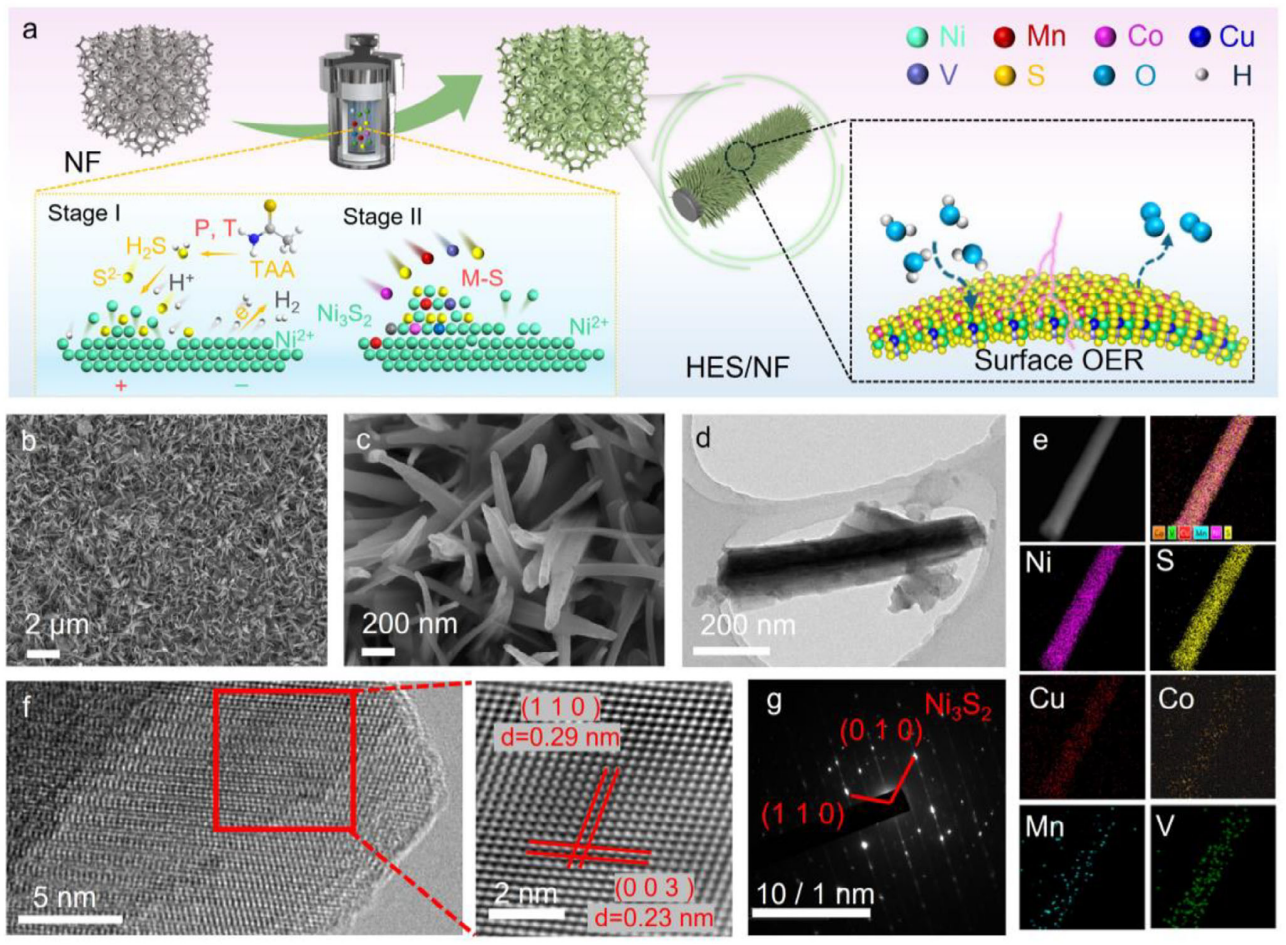
a) Schematic illustration of the construction of HES/NF and the corresponding oxygen evolution at the interface; b,c) Gradual enlarged magnification of SEM images; d) TEM image of the casual selected nanoneedle; e) HAADF‐STEM image and corresponding EDX maps; f) HRTEM image; g) SAED pattern.

The construction of the HES/NF via in situ corrosion process is illustrated in **Figure** **1**a, which involves a two‐step corrosion process: (1) Surface corrosion process: In this initial step, thioacetamide (TAA) is first decomposed into H_2_S under high‐temperature and high‐pressure conditions. This H_2_S subsequently undergoes hydrolysis to produce S^2−^ and H^+^ ions, creating a corrosive environment. During this phase, the NF corrodes preferentially at defective sites, acting as an anode, while other areas remain protected. (2) Corrosion‐deposition process: Once the H^+^ ions are mostly consumed, the corrosion process is restrained. At this point, heterometallic ions gradually combine with S^2−^ and deposited on the corrosion products on the surface of the NF, forming HES. This corrosion‐deposition process facilitates the incorporation of multiple metallic components, resulting in the formation of HES/NF. Gradual enlarged magnification of scanning electron microscopy (SEM) shown in Figure 1b,c demonstrated a dense array of nanoneedles across the NF surface, in which the nanoneedles have a diameter of ≈100 nm. Such morphology is beneficial to contribute abundant electrochemical active area for electrocatalytic reaction. To elucidate the physical structure at nanoscale, transmission electron microscopy (TEM) was employed. By casually selecting a nanoneedle (Figure 1d), the diameter was consistent with the SEM results (Figure 1c). The high angle annular dark field scanning TEM (HAADF‐STEM) image in Figure 1e with corresponding energy dispersive X‐ray spectroscopy (EDX) demonstrated highly uniform distribution of Ni, S, Co, V, Cu, Mn in a randomly selected nanoneedle. High‐resolution TEM (HRTEM) image (Figure 1f) showed distinct crossing lattice fringes with lattice spacings of 0.29 and 0.23 nm, corresponding to the (110) and (003) planes of rhombohedral structured Ni_3_S_2_, respectively. Selected area electron diffraction (SAED) analysis (Figure 1g) further substantiates the structure at an enlarged area, demonstrating the rich existing plane of Ni_3_S_2_ (110) and (010) along the nanoneedles. These findings preliminarily suggest that the nanoneedles, synthesized via in situ corrosion, primarily comprise high‐entropy sulfides with Ni_3_S_2_ as the predominant phase.

**Figure 2:**
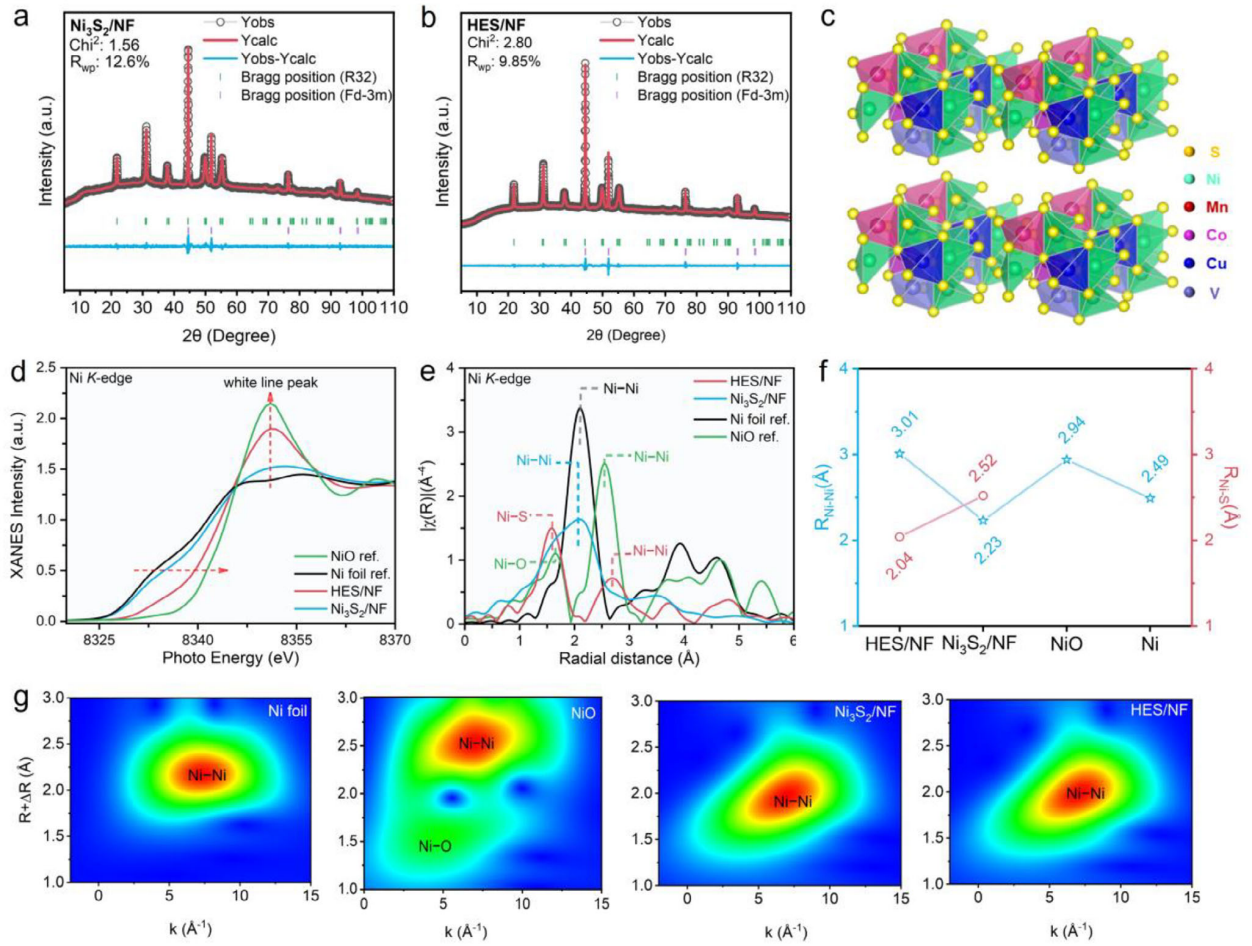
a,b) Refined XRD patterns of the Ni_3_S_2_/NF and HES/NF; c) Schematic crystal structure of HES/NF; d) Ni K‐edge XANES spectra; e) Fourier transformed k3‐weighted of Ni K‐edge EXAFS spectra; f) Corresponding dot and line chart of Ni‐Ni and Ni‐S bond length of different samples; g) WT spectra of different samples.

To determine the phase composition of the synthesized sulfides, X‐ray diffraction (XRD) was employed. The diffraction patterns of HES/NF, medium‐entropy sulfides (MES_T_/NF, MES_F_/NF), low‐entropy sulfides (LES/NF), and pure Ni_3_S_2_/NF samples (Figure S1, Supporting Information) display similar identical diffraction peaks, corresponding to a combination of standard reference patterns for elemental nickel (PDF#04‐0850) and Ni_3_S_2_ (PDF#44‐1418). This similarity suggests that the corrosion products at the surface of NF are predominantly Ni_3_S_2_ sulfides. Subsequent refinement of the XRD data, shown in **Figure** **2**a,b, allowed for quantitative analysis of the crystal structures of Ni_3_S_2_/NF and HES/NF. Both samples exhibit an orthorhombic crystal structure with space group R32. Notably, the R_wp_ and Chi^2^ values are within the ideal range, which further demonstrates their high phase purity. Based on this refined XRD data, a schematic model of the crystal structure of the HES/NF is proposed in Figure 2c, suggesting that other hetero metals randomly replaced some of the positions where nickel atoms occupied in the Ni_3_S_2_ structure.

To further examine electron structure evolution, like oxidation states, coordination environments between the two samples, and assess lattice distortion attributed to the high‐entropy effect, synchrotron radiation analysis was conducted. It can be seen from the Ni K‐edge spectra (Figure 2d), the white line peak of HES/NF is higher than the Ni_3_S_2_/NF. And the HES/NF sample exists a higher oxidation state of nickel species than the nickel species in Ni_3_S_2_/NF, implying enhanced reactivity conducive to the OER. Moreover, extended X‐ray absorption fine structure (EXAFS) analysis (Figure 2e), along with the corresponding R‐space and k‐space data (Figures S2 and S3, Supporting Information) reveal a weakened Ni‐Ni coordination environment in HES/NF, suggesting partial substitution of nickel by other hetero metals. These findings elucidate structural changes of HES/NF by introducing four types of hetero metals, which resulted in lattice distortion, and the weakened Ni‐Ni coordination, suggests changes in the local bonding environment. The longer Ni‐Ni bond distance in HES/NF, as compared to that in NiO, implies a higher oxidation state of Ni in HES/NF, which is beneficial to enhance the OER activity. In detail, as illustrated in Figure 2f and Table S1 (Supporting Information) the Ni‐Ni bond length in HES/NF (3.01 Å) exceeds the Ni_3_S_2_/NF (2.23 Å), providing additional evidence for lattice distortion induced by the high‐entropy effect. Furthermore, wavelet transform analysis (Figure 2g) reinforces the observed differences in the coordination environment. Collectively, these analyses offer a comprehensive understanding of the high‐entropy effect in influencing the electronic structure of HES/NF.

**Figure 3:**
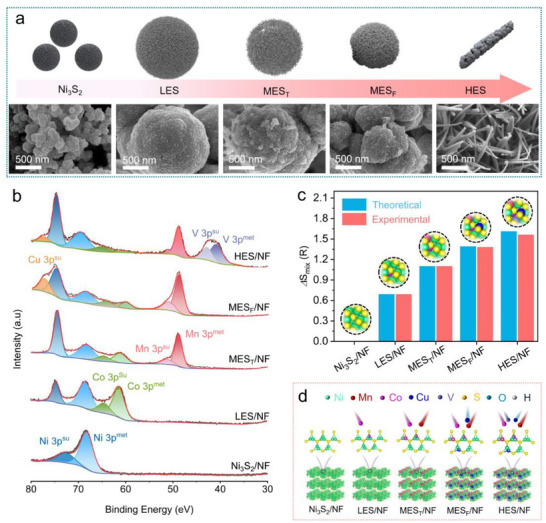
a) SEM images and the corresponding schematic illustrations and surface evolution by gradually increase of entropy levels as the sequence of Ni_3_S_2_/NF, LES/NF, MES_T_/NF, MES_F_/NF, and HES/NF; b) High resolution XPS spectra of Ni 3p, Co 3p, Mn 3p, Cu 3p, and V 3p; c) the *Δ*S_mix_ values of Ni_3_S_2_/NF, LES/NF, MES_T_/NF, MES_F_/NF, and HES/NF; d) Schematic illustration of the structural evolution mechanisms at increased entropy levels.

The surface evolution from Ni_3_S_2_/NF to gradual increase of entropy, and even to HES/NF was studied via SEM and X‐ray photoelectron spectroscopy (XPS). As depicted in **Figure** **3**a, the Ni_3_S_2_/NF primarily appears as ≈200 nm‐diameter of microspheres with rough‐surface. Upon gradual increase of hetero metals, thread‐like nanomorphology on the microspheres can be clarified in LES/NF, then obvious evolution of nanowires on the many microspheres from MES_T_/NF to MES_F_/NF. Besides, the sphere diameter showed gradual decrease from Ni_3_S_2_/NF to MES_F_/NF and even disappeared for HES/NF. The dissolution of microsphere is likely to participated in the growth of dense vertical distribution of nanoneedles. The survey XPS spectrum (Figure S4, Supporting Information) of the sulfides confirms the presence of the corresponding metal species, reflecting the successful incorporation of hetero raw materials. The high‐resolution Ni 2p XPS spectra (Figure S5, Supporting Information) of HES/NF, MES_F_/NF, MES_T_/NF, LES/NF and Ni_3_S_2_/NF exhibit characteristic peaks for Ni^0^ and Ni^δ⁺^ (Ni^2+^/Ni^3+^) at binding energies of 852.8 eV (Ni^0^),^[^ ^40^ ^]^ 855.8 eV (Ni^2+^),^[^ ^41^ ^]^ and 857.1 eV (Ni^3+^),^[^ ^42^ ^]^ along with a satellite peak at ∼861.8 eV. The Ni^0^ signal originates from the metallic NF substrate, which facilitates electron transfer.^[^ ^41^ ^]^ With increasing configurational entropy, the intensity of Ni species progressively decreases, while the S intensity increases (Figure S6, Supporting Information), indicating that surface Ni is gradually replaced by other heterometals to form M‐S bonds. This aligns well with the in situ corrosion schematic in Figure 1a. As shown in Figures S7–S10 (Supporting Information), the Co 2p spectrum features peaks at 778.8 (Co^2+^), 783.1 (Co^3+^), and 787.0 eV (satellite peak),^[^ ^43^, ^44^ ^]^ while the Mn 2p spectrum exhibits peaks at 640.7 (Mn^2+^) and 645.6 eV (Mn^3+^).^[^ ^45^ ^]^ The fitted Cu 2p spectra display peaks at 932.2 (Cu^+^) and 933.8 eV (Cu^2+^).^[^ ^46^, ^47^ ^]^ Similarly, the V 2p spectrum of HES/NF shows peaks at 517.2 (V^4+^) and 515.7 eV (V^3+^).^[^ ^48^ ^]^ To avoid the issue of Auger transition peak overlap among different elements in high‐entropy materials, 3p signals of transition metals were further investigated.^[^ ^49^ ^]^ As shown in Figure 3b, within the 30.0–80.0 eV range, Ni 3p was deconvoluted into Ni‐S (74.7 eV, Ni 3p^su^) and Ni‐Ni (69.8 eV, Ni 3p^met^) bonds.^[^ ^50^ ^]^ Similarly, Co 3p appeared at 64.4 eV (Co 3p^su^) and 61.2 eV (Co 3p^met)^, Mn 3p at 51.3 eV (Mn 3p^su^) and 48.8 eV (Mn 3p^met^), and V 3p at 42.6 eV (V 3p^su^) and 40.8 eV (V 3p^met^), with a Cu peak at 77.4 eV (Cu 3p^su^).^[^ ^49^ ^]^ These results align well with the XPS 2p analysis. It is hypothesized that as the gradual increase of entropy, the valence states of the catalysts showed negligible change, but promoted the M^δ⁺^ toward higher binding energy shifts, resulted in the regulation of surface electronic structure.

To specifically determine the entropy levels of the sulfides, a rational configurational entropy formula (Equation S1, Supporting Information) was adopted, which includes the gas constant 𝑅 as a factor and depends on the mole fractions of different metallic elements in the cation sublattice. The configurational entropy (*Δ*𝑆_mix_) calculated by both theoretically atomic ratio of feed during sulfides preparation and practical ratios of heterometals determined via Inductively Coupled Plasma Optical Emission Spectrometry (ICP‐OES, Table S2, Supporting Information). According to previous report, the *Δ*𝑆_mix_ values are categorized as follows: high entropy for *Δ*𝑆_mix_≥1.5𝑅, medium entropy for 1.0𝑅≤*Δ*𝑆_mix_ <1.5𝑅, and low entropy for *Δ*𝑆_mix_ <1.0𝑅.^[^ ^51^ ^]^ As illustrated in Figure 3c and Table S2 (Supporting Information), the *Δ*𝑆_mix_ of HES/NF exceeds 1.5R (1.60R), that of MES/NF falls within the range of 1R to 1.5R (1.10R, 1.39R), and that of LES/NF is less than 1R (0.69R). These results confirm that the synthesized heazlewoodite catalyst materials satisfy the definitions of high‐, medium‐, and low‐entropy materials. According to the microstructure evolution and electronic structure regulation with varying entropy states, a schematic representation of surface structural evolution can be drawn in Figure 3d, especially from microsphere to nanoneedle transitions and the electronic optimizations with increasing entropy levels. This transformation will play significant impacts for catalytic activity underscoring the critical role of entropy engineering.

**Figure 4:**
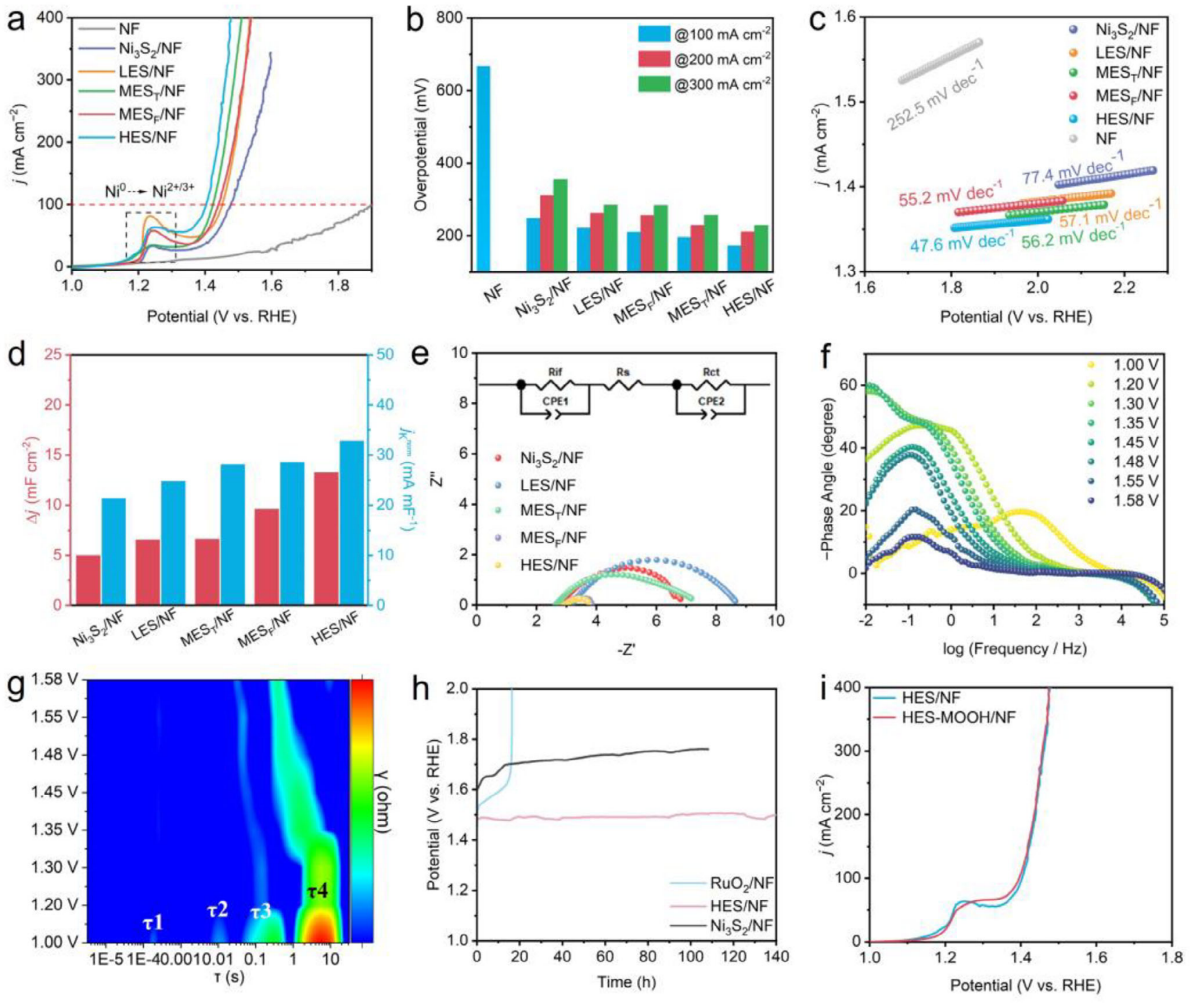
a) Polarization curves of the Ni_3_S_2_/NF, LES/NF, MES_T_/NF, MES_F_/NF, HES/NF, and NF; b) Comparison of overpotential at constant current densities of 100.0, 200.0, and 300.0 mA cm^−2^; c) The corresponding Tafel slopes; d) Histograms of *C*_dl_ at different scan rates and *j*_K_^norm^ values at potential of 1.48 V; e) Nyquist plots; f,g) In situ impedance testing diagram and its relaxation time distribution diagram; h) Chronopotentiometric curves of Ni_3_S_2_/NF, HES/NF and RuO_2_/NF at constant current density of 100.0 mA cm^−2^; i) Polarization curves of HES/NF and HES‐MOOH/NF.

The electrocatalytic performance toward OER was studied using a three‐electrode system. As shown in **Figure** **4**a, the HES/NF catalyst demonstrates outstanding OER catalytic activity compared to other catalysts with lower entropy. Specifically, a small overpotential of 172.0 mV at a current density of 100.0 mA cm^−2^ was achieved for HES/NF (Figure 4b), significantly lower than that of MES_F_/NF (195.0 mV), MES_T_/NF (210.0 mV), LES/NF (221.0 mV), Ni_3_S_2_/NF (248.0 mV), and NF (668.0 mV). Same conclusions can be drawn at higher current density of, even 200.0 and 300.0 mA cm^−2^, demonstrating the high potentially application of HES/NF at extreme conditions. Tafel plots derived from the polarization curves in Figure 4a were plotted (Figure 4c) to study the reaction kinetics. After fitted, the Tafel slope of HES/NF (47.6 mV dec^−1^) is lower than that of MES_F_/NF (55.2 mV dec^−1^), MES_T_/NF (56.2 mV dec^−1^), LES/NF (57.1 mV dec^−1^), Ni_3_S_2_/NF (77.4 mV dec^−1^), and NF (252.5 mV dec^−1^), indicating that the incorporation of additional transition metals enhances the intrinsic catalytic activity. Since electrochemical active surface area (ECSA) is a key parameter both impacting the OER activity and reaction kinetics. Given that ECSA is proportional to the double‐layer capacitance (*C*_dl_) under non‐Faradaic potential windows, the *C*_dl_ values were obtained from cyclic voltammetry (CV) curves at a potential window of 0.60–0.70 V at varying scan rates (Figure S11, Supporting Information). Besides, the current densities derived from the LSV curves in Figure 4a at kinetic potential of 1.48 V were divided by the corresponding *C*_dl_ values, which is denoted as the normalized kinetic current density (*j*_K_^norm^), indicating the intrinsic activity evolution with increasing entropy levels. As shown in in Figure 4d, the HES/NF demonstrated the highest *C*_dl_ and *j*_K_^norm^ values of 13.2 mF cm^−2^ and 32.8 mA mF^−1^, relative to MES_F_/NF (9.6 mF cm^−2^ and 28.58 mA mF^−1^), MES_T_/NF (6.7 mF cm^−2^ and 28.1 mA mF^−1^), LES/NF (6.6 mF cm^−2^ and 24.8 mA mF^−1^), and Ni_3_S_2_/NF (5.0 mF cm^−2^ and 21.3 mA c mF^−1^), which together contribute to its superior OER activity.

Electrochemical impedance spectroscopy (EIS) measurements were performed to investigate in‐depth electrocatalytic kinetics. Figure 4e shows Nyquist plots of catalysts with increasing entropy. After fitted via the equivalent circuit (inset), HES/NF exhibits the lowest charge transfer resistance (R_ct_) of 3.7 Ω compared to MES_F_/NF (3.9 Ω), MES_T_/NF (6.8 Ω), LES/NF (8.6 Ω), and Ni_3_S_2_/NF (7.2 Ω), indicating that the high‐entropy designment enhances electron transfer. To further understand the dynamic electrochemical reaction kinetics of HES/NF, in situ impedance measurements were conducted to assess reactant adsorption and desorption kinetics on the electrode surface. Based on prior studies,^[^ ^52^ ^]^ we find that the high‐frequency (HF) region is associated with internal oxidation within the electrode, whereas the low‐frequency (LF) region corresponds to charge imbalances caused by oxide species at the electrode interface. As shown in Figure 4f, below 1.2 V versus RHE, the HF region exhibits notable changes, suggesting electrocatalytic oxidation, while above 1.2 V versus RHE, a pronounced decrease in the LF phase angle signals the onset of OER. HES/NF demonstrates both a faster electrooxidation rate and OER kinetics than other catalysts (Figure S12, Supporting Information), indicating that its unique high‐entropy structure, enriched with nanoneedles providing a large surface area and abundant active sites, effectively facilitates charge and mass transfer, thus enhancing OER performance. Accordingly, the HES catalyst demonstrated excellent OER properties which surpass most of those reported catalysts (Table S3, Supporting Information). To further probe the dynamic reaction mechanism of HES/NF at different OER potentials, relaxation time analysis was performed via the in situ EIS process, as shown in Figure 4g, where four characteristic peaks were identified: τ1, τ2, τ3 and τ4, where τ1 represents the contact impedance, τ2 reflects the adsorption of H_2_O on the catalyst surface active sites and disappears close to 1.23 V, and τ3, τ4 represent the charge transfer impedance (R_ct_). As demonstrated, the R_ct_ starts to shift ≈ 1.4 V, which is also consistent with the overpotential of this catalyst; Notably, τ1 and τ2 rapidly disappear after the OER begins, while τ3 and τ4 gradually decrease and shift, particularly τ3, which shows a significant change at 1.3 V versus RHE. This observation suggests remarkable surface structural evolution occurring on the catalyst during the reaction.

Beyond OER activity, working durability is also essential for practical water‐splitting applications. The stability of Ni_3_S_2_/NF, HES/NF and commercial RuO_2_/NF were evaluated at a constant current density of 100.0 mA cm^−2^ through chronopotentiometry technique, in which HES/NF displayed outstanding stability, showing minimal potential change even after 140.0 h, while commercial RuO_2_ exhibited a sharp increase in potential after only 16.7 h (Figure 4h). Additionally, Ni_3_S_2_/NF initially exhibited a high potential of 1.58 V, which gradually increased to ≈1.72 V after 100 h, indicating poor OER stability. Moreover, the OER polarization curves of HES before and after chronopotentiometry tests is compared in Figure 4i. Notably, the OER activity showed negligible decay even after a 140‐h stability test, especially at extreme conditions, like at current density of 400 mA cm^−2^. Additionally, the obvious oxidation peak at approximately 1.25 V evolved to a wide plain peak, corresponding to the surface specie evolution mainly from Ni_3_S_2_ to NiOOH.^[^ ^53^ ^]^

**Figure 5:**
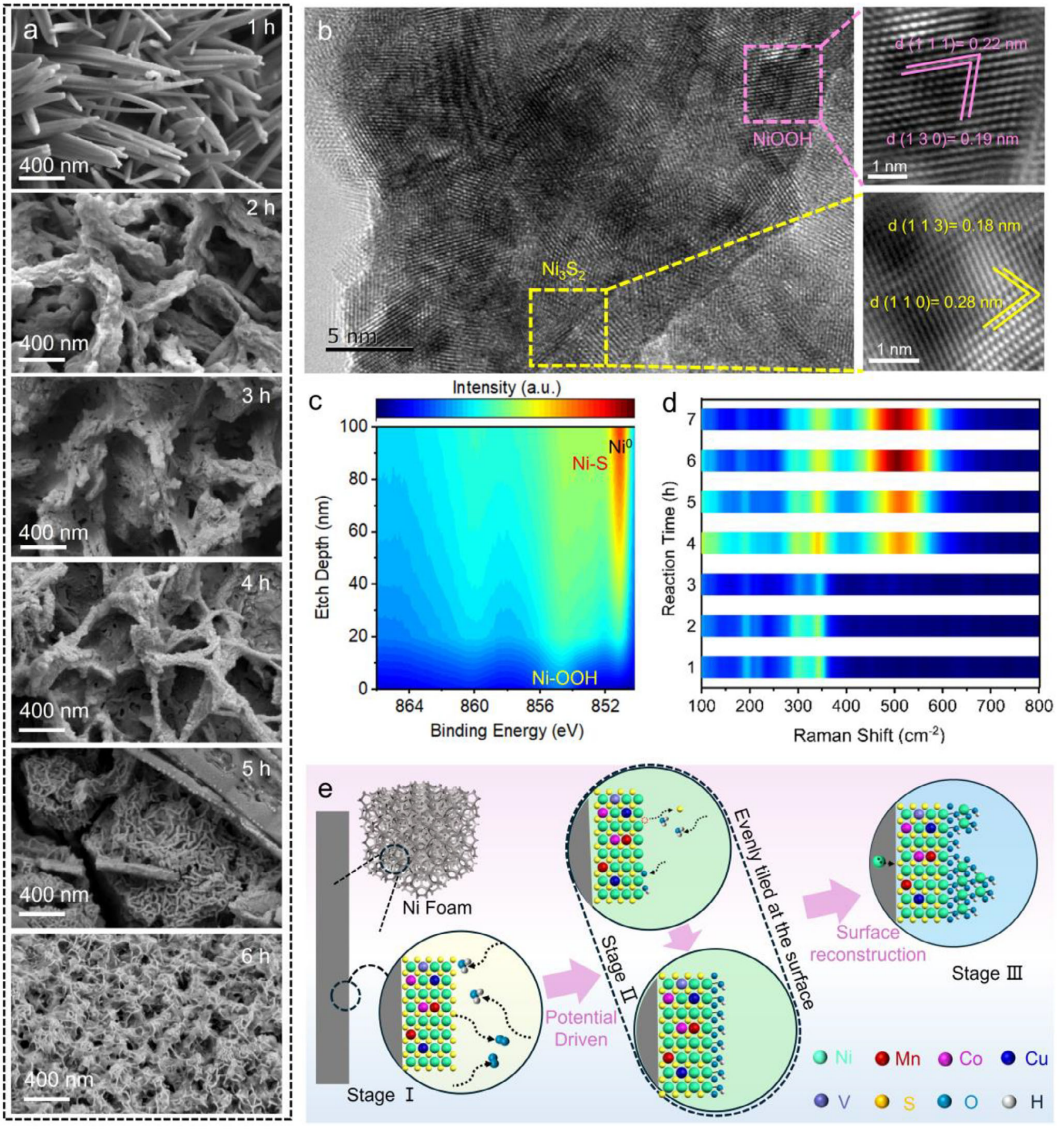
a) SEM images of the HES/NF operated at increased time from 1 to 6 h; b) The HRTEM and the SAED pattern of the HES/NF after 6 h reaction; c) The high resolution XPS spectra of Ni 2p at difference depth profiling; d) The ex situ Raman spectra of the HES/NF at increased time from 1 to 6 h; e) Schematic illustration of the surface reconstruction mechanisms of HES/NF.

To further investigate the dynamic evolution of high‐entropy sulfide catalysts during the OER, ex situ SEM characterization was conducted. Catalysts were collected at different reaction times (1, 2, 3, 4, 5, and 6 h) during chronoamperometry testing. As shown in **Figure** **5**a, new surface structures began to form on the nanoneedles over time, gradually transforming from nanoneedles into sheets. Specifically, at 2^nd^, 3^rd^, and 4^th^ h, dendritic features developed on the nanoneedle surfaces, causing nearby nanoneedles to aggregate. At the 5^th^ and 6^th^ h reaction times, the structures had evolved into nanosheets and even nanoflower‐like architectures. The evolved products after 6 h chronoamperometry test were examined by TEM. As shown in Figure S13 (Supporting Information), the catalyst predominantly exhibits sheet morphology. HRTEM images (Figure 5b) revealed two distinct orientations of lattice fringes at the inner part with adjacent lattice spacings of 0.28 and 0.18 nm, corresponding to the (110) and (113) planes of rhombohedral Ni_3_S_2_. The newly formed phase at the surface showed adjacent lattice spacings of 0.22 and 0.19 nm, corresponding to the (111) and (130) planes of NiOOH. SAED pattern (inset) at the same position also demonstrated the co‐existence phases of both Ni_3_S_2_ and NiOOH. EDX maps (Figure S14, Supporting Information) indicated a notable increase in oxygen content in the post‐reaction sample and a decrease in sulfur compared to the initial HES/NF, while the distribution of other metals remained relatively unchanged. This suggests that HES/NF undergoes a dynamic transformation at the electrochemical interface with morphology and phase evolution during the OER. For comparison, after a 6‐h stability test, the Ni_3_S_2_/NF revealed a transition from the original microspheres to a rough, bulk surface (Figure S15, Supporting Information), suggesting an unstable electrocatalytic interface under oxidation‐driven potential. To further confirm this surface evolution, in situ XPS depth‐profiling analysis was conducted (Figure 5c; Figure S16, Supporting Information). The high‐resolution Ni 2p profile revealed a gradual transition from oxygen‐related states to sulfides with increasing etch depth. High‐resolution O 1s spectra (Figure S16a, Supporting Information) identified two dominant peaks at 531.9 and 529.9 eV, corresponding to M─OOH and M─O bonds, respectively. As the profiling depth increased, these peaks shifted to lower binding energies with decreasing intensity, indicating the disappearance of M─OOH bonds. Similarly, XPS depth profiling of S 2p (Figure S16b, Supporting Information) showed a negligible Ni─S bond before etching, with its intensity gradually increasing as etch depth increased. Additionally, ex situ Raman spectroscopy was also performed to study the evolution of the HES/NF and Ni_3_S_2_/NF at the electrochemical interface, as shown in Figure 5d and Figure S17 (Supporting Information). A detailed record of peaks shifts with the increasing of reaction time. Initially, the high‐entropy sulfide exhibits a characteristic peak at 290 cm^−1^, which progressively transformed to 475 and 555 cm^−1^ with increasing potential, corresponding to the E_9_ bending vibration and A_19_ stretching vibration modes of M–O in MOOH (Ni–O in NiOOH for Ni_3_S_2_/NF), respectively.^[^ ^54^, ^55^ ^]^ Such evolution efficiently addressed the poor phase stability at high oxidation potentials by evolving stabilized MOOH species, offering a new perspective for developing efficient OER electrocatalysts.

Accordingly, the electrochemical interface transformation of HES driven by high oxidation potential can be described as a three‐stage process, as illustrated in Figure 5e. During the initial stage, the HES interface generates a large amount of oxygen‐containing free radicals during the OER process, leading to the adsorption of *OOH species on the catalytic surface. Subsequently, in the intermediate stage, some of the adsorbed *OOH species form a MOOH hetero interface, which begins to cover the surface of HES. As the OER process continues into the final stage, the MOOH accumulates to form a stable high‐entropy hydroxide‐sulfide composite (HES‐MOOH/NF). This reconstructed heterogeneous interface significantly enhances catalytic stability while maintaining high catalytic activity and suppressing HES deactivation.

**Figure 6:**
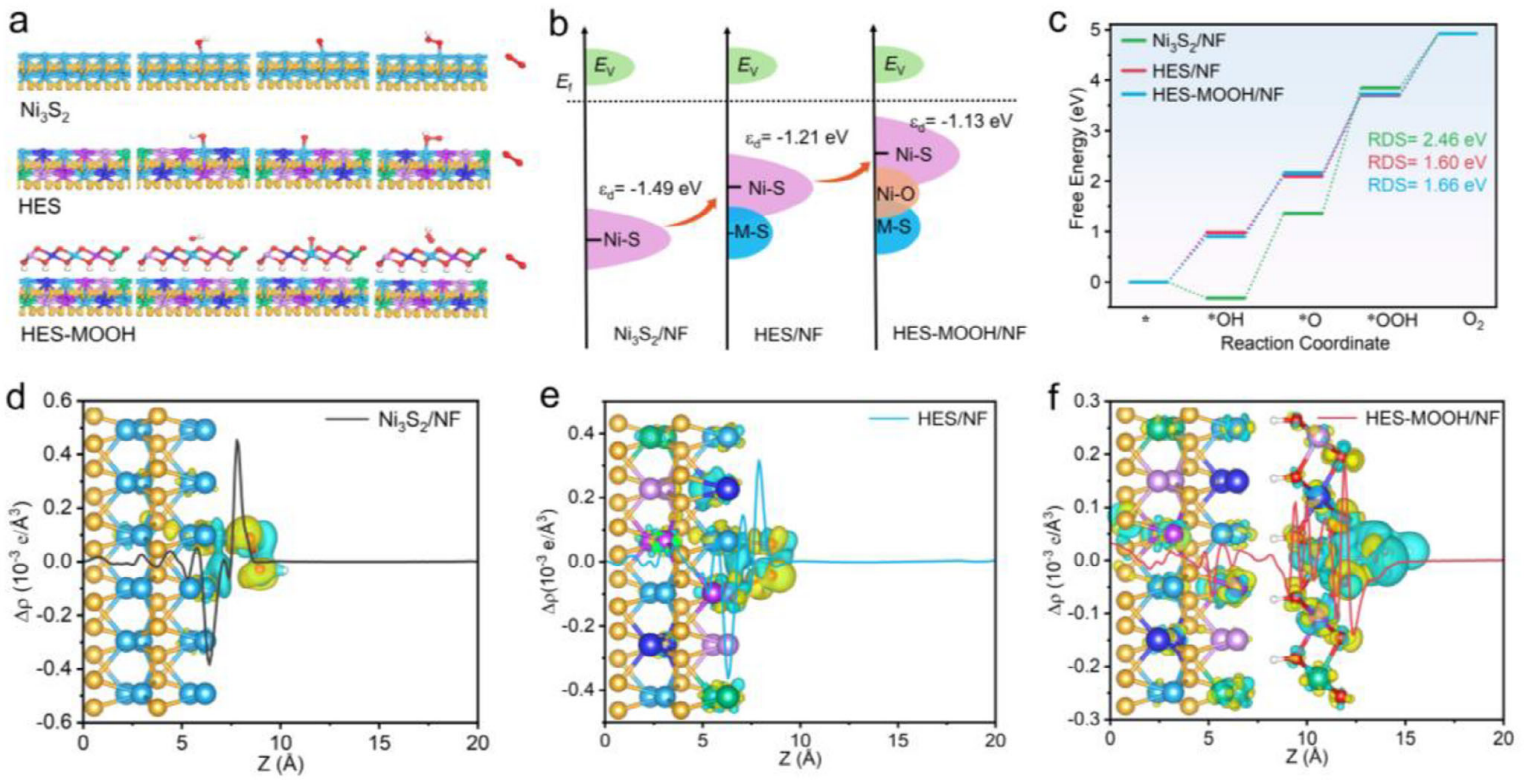
a) Theoretical models of the optimized configurations of Ni_3_S_2_, HES and HES‐MOOH and corresponding water dissociation process; b) d‐band centers of different sulfides; c) water dissociation free energy change profile of different sulfides during OER process; Charge density difference and planar‐averaged electron density difference of the Ni_3_S_2_/NF d), HES/NF e) and HES‐MOOH/NF f).

Density functional theory (DFT) calculations were performed to assess the electronic states, transition states, and adsorption‐free energy evolution of Ni_3_S_2_/NF, HES/NF, and HES‐MOOH/NF. Theoretical models of the optimal configurations with stepwise adsorption models for the oxygen evolution were depicted in **Figure** **6**a. A schematic representation of the d‐band centers for Ni_3_S_2_/NF, HES/NF, and HES‐MOOH/NF (Figure 6b) was plotted based on the density of states (DOS) profile shown in Figure S18 (Supporting Information). As can be seen, the d‐band center (*ɛ*_d_) of HES/NF is −1.21 eV, compared to −1.49 eV for Ni_3_S_2_/NF. This indicates that the formation of a high‐entropy system facilitates the upward shift of *ɛ*_d_ toward the Fermi level (*E*_f_), enhancing OH* adsorption on the HES/NF surface. This improvement is attributed to the incorporation of heterometals, which form a high‐entropy catalytic system. After interface transformation driven by oxidation potential, the *ɛ*_d_ of HES‐MOOH/NF shifts further upward to −1.13 eV. This shift suggests a reduction in electron density in antibonding states and an enhancement in the adsorption strength toward oxygen‐containing intermediates.^[^ ^53^, ^56^ ^]^

Free energy calculations were performed to further analyze the catalytic process, as depicted in the reaction energy profiles. The generation of OOH* was identified as the rate‐determining step (RDS) of the overall reaction. During the OH* adsorption step, the free energy change *ΔG*_OH_* for Ni_3_S_2_/NF was calculated to be 1.68 eV (Figure 6c), substantially higher than that for HES/NF (*ΔG*_OH_* = 1.12 eV). This indicates a significantly reduced reaction energy barrier due to the introduction of hetero metals and the high‐entropy catalytic structure. Additionally, the *ΔG*_OH_* for HES‐MOOH/NF was found to be 1.26 eV, comparable to that of HES/NF, suggesting that the surface evolution from HES to HES‐MOOH has minimal impact on OER activity. In the RDS, HES/NF and HES‐MOOH/NF demonstrated reduced free energy values of 1.60 and 1.66 eV, respectively, both significantly lower than that of Ni_3_S_2_/NF. This indicates the higher electrocatalytic activity of HES/NF for OER. Furthermore, as shown in Figure 6d–f, the charge distribution analysis highlights uniform charge redistribution within HES/NF due to the introduction of hetero metals, reflecting substantial electronic structure evolution with the high‐entropy structure. Notably, the charge transfer from HES to HES‐MOOH during the surface evolution of HES/NF contributes to the final HES‐MOOH/NF structure. Combined with the free energy calculations, this charge redistribution enhances electrocatalytic stability without compromising OER activity.^[^ ^57^ ^]^

**Figure 7:**
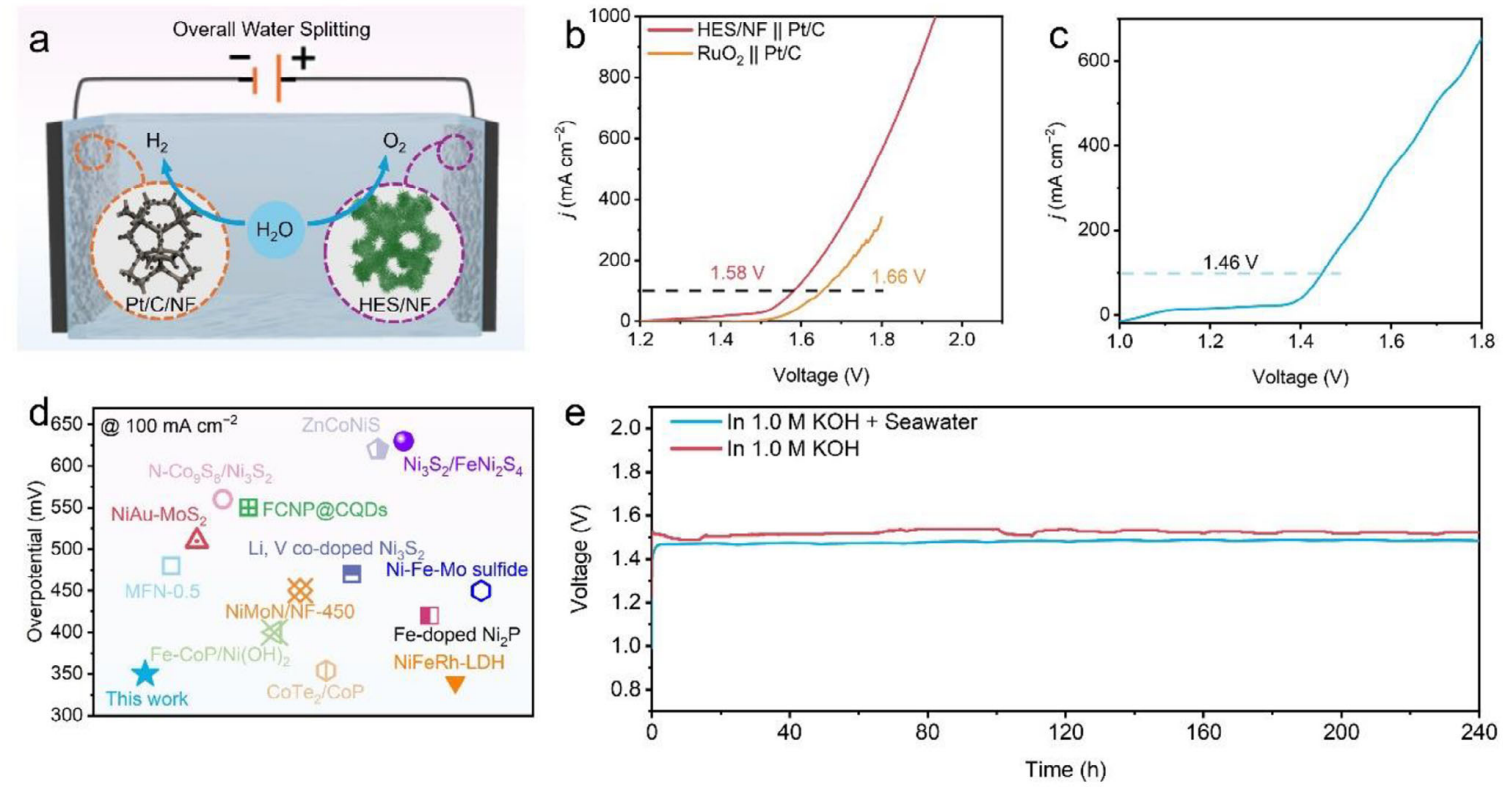
a) Schematic water splitting diagram; b) Polarization curves of HES/NF‖Pt/C and RuO_2_‖Pt/C couples for overall water splitting; c) Polarization curves of Pt/C//HES/NF for overall water splitting in seawater conditions; d) Comparison chart of overpotentials for overall water splitting; e) Chronopotentiometry curves at constant current density of 100.0 mA cm^−2^ in different electrolytes.

Considering the remarkable OER catalytic performance of HES/NF, a simple water electrolyzer configuration was assembled to investigate the practical water splitting (**Figure** **7**a). In this configuration, HES/NF, Pt/C‐coated NF, and 1 m KOH solution were employed as the anode, cathode, and electrolyte respectively. The polarization curve (Figure 7b) demonstrated excellent water splitting activity of HES/NF with a small voltage of 1.58 V at a high driven current density of 100.0 mA cm^−2^. Additionally, stability testing was conducted at the same current density value using chronopotentiometry test. After 240 h of continuous durability test, negligible potential change occurred (Figure 7e), highlighting the long‐term stability and practical viability of the HES/NF system for electrochemical water splitting. To further evaluate the practical application of HES/NF, the OER tests were conducted in simulating seawater environment. As illustrated in Figure 7c, HES/NF achieved a small cell voltage of just 1.46 V driven by a high current density of 100.0 mA cm^−2^. The voltage required for overall water splitting is significantly lower than that of most reported self‐supported electrode materials (Figure 7d; Table S4, Supporting Information), demonstrating that the designed HES/NF functions as an efficient and highly effective bifunctional electrocatalyst. Corresponding stability test (Figure 7e) revealed that the HES/NF system maintained a stable potential over 240 h of continuous operation, showcasing its robustness and potential for practical applications in electrolytic seawater.

## 3 Conclusion

In summary, we successfully addressed the challenges associated with entropy modulation and the dynamic reconfiguration of high‐entropy sulfides during the OER. The innovative in situ corrosion strategy that efficiently transformed low‐valent NF substrate into HES/NF, significantly enhanced the OER activities. Notably, HES/NF achieved remarkably low overpotentials of 172.0 mV at 100.0 mA cm^−2^ and 229.0 mV at an extreme current density of 300.0 mA cm^−2^, underscoring its superior catalytic efficiency. A series of heazlewoodite catalysts with varying levels of entropy demonstrated a direct correlation between entropy and electrocatalytic activity. Comprehensive suite of in situ and ex situ characterization techniques demonstrated that HES/NF dynamically transformed into a stable sulfide‐hydroxide oxidation species interface under OER conditions. This transition, coupled with lattice distortion, optimized the electrostatic potential distribution, thereby ensuring superior catalytic activity while preventing surface sulfide deactivation through the formation of stable HES‐MOOH species. DFT calculations further elucidated the adsorption strength of oxygen‐containing intermediates on Ni_3_S_2_/NF and HES/NF before and after surface reconstruction, further verified the charge redistribution affected electrocatalytic stability without compromising OER activity. Benefiting from the evolution of HES‐MOOH at the surface, excellent OER activities, stability, and water‐splitting performance when paired with a Pt/C cathode were achieved. Overall, this work provided deeper insights into the mechanisms governing the relationship between entropy, surface reconstruction, and electrocatalytic activity.

## 4 Experimental Section

### Preparation of HES/NF Catalyst

The NF substrate is pretreated before use. Specifically, the NF is cut into sizes of 3 × 4 cm and then soaked in a 0.1 m HCl solution for 10 min to remove surface impurities. It is then alternately rinsed three times with deionized water and ethanol, followed by drying in a vacuum oven at 60 °C. Under magnetic stirring, 20 mmol of thioacetamide, 2 mmol of nickel chloride, 2 mmol of copper chloride, 2 mmol of manganese chloride, 2 mmol of cobalt chloride, and 2 mmol of ammonium metavanadate are completely dissolved in a mixed solution of 30 mL anhydrous ethanol and DMF in a volume ratio of 1:1. The solution is then placed in a reaction kettle along with the NF substrate and reacted at 160 °C for 8 h. Finally, the NF substrate is removed and alternately washed several times with deionized water and anhydrous ethanol, and dried for 10 h at 60 °C, resulting in HES/NF. To study the effect of different entropy states on the catalyst itself, MES_F_/NF (Ni, Co, Cu, Mn), MES_T_/NF (Ni, Co, Mn), LES/NF (Ni, Co), and Ni_3_S_2_/NF (Ni Foam) were synthesized under the same conditions. The only variable that changed was the number of metals added, while the total amount of substance was the same (10 mmol of metal salts).

### Physical Characterization

XRD information was collected by using a Bruker D8 Advance diffractometer with Cu Kα radiation (λ = 1.5406 Å). The morphologies and microstructures of the as‐prepared samples were characterized by a field‐emission SEM (FESEM, ZEISS300) instrument and a TEM instrument (JEOL JEM−2100F). The compositional information was characterized by using a Raman instrument (Thermo DXR2xi). The valence state information was characterized by using an XPS instrument using 150 W Al Kα radiation as an excitation source. The measurements were made on the K‐Alpha spectrometer and all binding energies were calibrated by the C 1s peak at 284.8 eV. The XPS 3p spectra were measured within a range of 25.0 to 80.0 eV, under the same testing conditions. The X‐ray absorption fine structure (XAFS) spectra of Ni K‐edge of HES/NF and Ni_3_S_2_/NF were collected in transmission mode on Table XAFS‐500 (Specreation Instruments Co., Ltd.) at 25 kV and 20 mA. The Si (551) spherically bent crystal analyzers with a radius of curvature of 500 mm were used. Before the test, the samples were grinded and tableted into slices with a diameter of 12.7 mm. XPS depth profiling experiments were conducted using a Thermo Scientific K‐Alpha instrument (Thermo Fisher). An Ar ion beam with an etch rate of 0.2 nm s^−1^ over a 2 × 2 mm^2^ area, and the etching energy was set to 1000 eV.

### Electrochemical Measurements

All the electrochemical measurements were performed by adopting a CHI‐760E electrochemical workstation and a three‐electrode system. 1.0 m KOH aqueous solution was used as electrolyte, and the as‐prepared catalysts, platinum electrode, and reversible hydrogen electrode (RHE) were used as working electrode, counter electrode, and reference electrode, respectively. Before electrochemical testing, oxygen must be injected into the electrolyte until it is saturated to maintain the OER process oxygen balance in water. Cyclic voltammetry (CV) was run at a scan rate of 50 mV s^−1^, and the potential range was 0.05–1.2 V (vs RHE). The scanning cycle voltammetry (LSV) voltage range is selected from 0.8–1.8 V, and the scanning rate is 5 mV s^−1^. The line of Tafel is obtained by fitting the LSV curve. EIS is tested at a potential of 1.5 V (vs RHE), the rate ranges from 0.01 to 100 000 Hz and the amplitude values were set to be 5 mV. The *C*_dl_ was evaluated from the CV curves measured in the range of 5−200 mV s^−1^.

Alkaline water and seawater splitting experiments were conducted in a self‐made two‐electrode system, with HES/NF serving as the anode and Pt/C as the cathode. The chronoamperometry and chronopotentiometry tests were conducted using current densities of 100 mA cm^−2^ in both 1.0 m KOH solution and 1.0 m KOH + seawater solution, respectively.

## Conflict of Interest

The authors declare no conflict of interest.

## Supporting information



Supporting Information

## Data Availability

The data that support the findings of this study are available from the corresponding author upon reasonable request.
