# Supplementary material for: Enhancing Oxygen Evolution Electrocatalysis in Heazlewoodite: Unveiling the Critical Role of Entropy Levels and Surface Reconstruction
Source: Adv Mater. 2025 Apr 7;37(21):2501186. doi: 10.1002/adma.202501186 (PMC12107225; doi:10.1002/adma.202501186)
Supplement: Supplementary file 1 — Supporting Information [file ADMA-37-2501186-s001.docx]

**Supporting Information**

**Enhancing Oxygen Evolution Electrocatalysis in Heazlewoodite: Unveiling the Critical Role of Entropy Levels and Surface Reconstruction**

Hangning Liu^+^, Xinghang Liu^+^, Anbang Sun^+^, Cuijuan Xuan*, Yingjun Ma, Zixuan Zhang, Hui Li, Zexing Wu*, Tianyi Ma*, and Jie Wang*

H. Liu, X. Liu, Y. Ma, Prof. C. Xuan, Prof. J. Wang

Qingdao Engineering Research Center of Agricultural Recycling Economy Materials,

College of Chemistry and Pharmaceutical Sciences,

Qingdao Agricultural University,

Qingdao 266109, P. R. China.

E-mail: [wangjie@qau.edu.cn](mailto:wangjie@qau.edu.cn); cjxuan@qau.edu.cn

H. Liu, Z. Zhang

School of Industrial and Information Engineering,

Politecnico di Milano,

Milano 20133, P. R. Italia.

A. Sun

Shandong Institute of Non-Metallic Materials

Jinan 250031, China.

H. Li, T. Ma

Centre for Atomaterials and Nanomanufacturing (CAN)

RMIT University,

Melbourne, VIC 3000, Australia

Email: tianyi.ma@rmit.edu.au

Prof. Z. Wu

State Key Laboratory Base of Eco-chemical Engineering,

College of Chemistry and Molecular Engineering,

Qingdao University of Science & Technology,

53 Zhengzhou Road,

266042 Qingdao, P. R. China.

Email: splswzx@qust.edu.cn

^+^The authors contributed equally to this work.

**Experimental Section**

***DFT part:*** Density functional theory (DFT) based first-principles calculations are conducted using projected augmented wave (PAW) method implemented in the Vienna ab initio simulation package (VASP). The Kohn-Sham one-electron states are expanded using the plane-wave basis set with a kinetic energy cutoff of 450 eV. The generalized gradient approximation (GGA) with Perdew-Burke-Ernzerhof (PBE) was applied to address the nonlocal exchange correlation energy. A sufficiently large vacuum region of 15 Å in the vertical that was used for the systems to investigate the mechanism of surface reactions. The Brillouin-zone integration is carried out using the Monkhorst-Pack sampling method with a density of 2×3×1 for optimizations. During the geometry optimizations, it was allowed to relax until the maximum magnitude of the force acting on the atoms is smaller than 0.02 eV Å^−1^, and the total energy convergence criterion is set to 1 × 10^-4^ eV. The calculation of the Gibbs free energy of the intermediates followed the Nørskov method. The adsorption energy (*E*_ads_) of the activated molecules on electrocatalyst surfaces was defined as: *ΔE*_ads_ = *E*_ad/sub_ - *E*_ad_ - *E*_sub_. The *E*_ad/sub_, *E*_ad_, and *E*_sub_ represent the energies of the substrate with adsorbate species, the adsorbate species and the substrate, respectively. For each step, Gibbs free energy(*ΔG*) change was calculated as: *ΔG* =*ΔE*_ads_ + *Δ*ZPE – T*Δ*S. The ΔZPE, *Δ*S and T represent zero-point energy change, entropy change between the adsorbate species and the substrate, and the temperature (The temperature is set to 298.15 K in this work), respectively. *Δ*ZPE can be calculated by additional vibrational frequency analysis.

The configurational entropy (*Δ*𝑆_mix_) can be calculated using Equation (1):

$S_{\left\{ mix \right\}}= -R \sum x_{i}ln x_{i}$ (1)


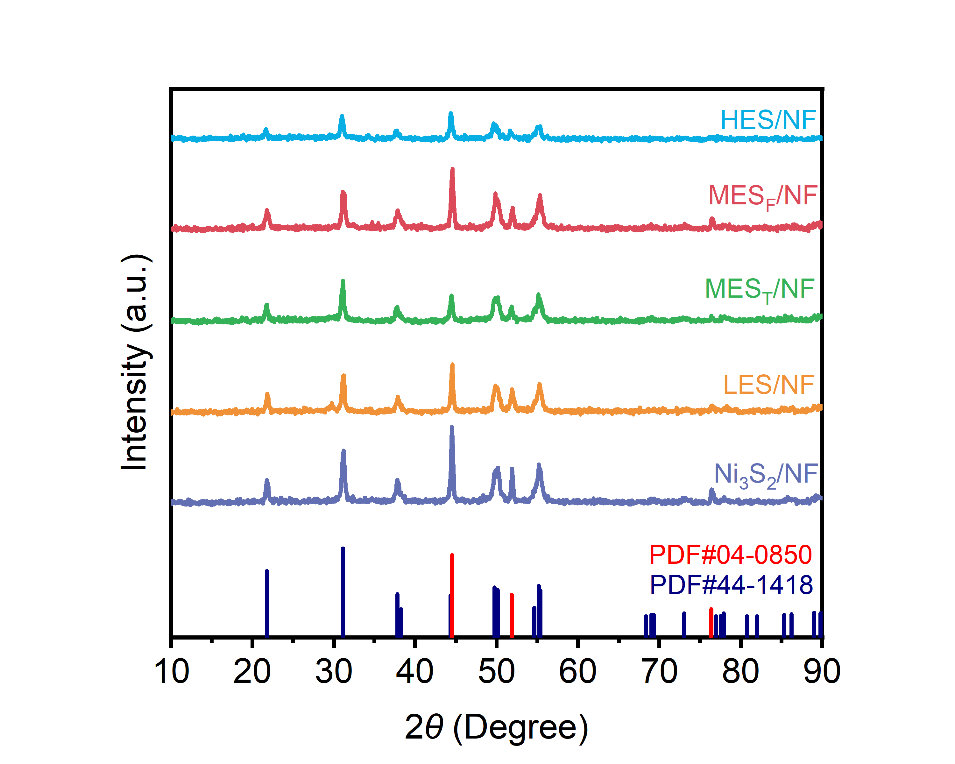


**Figure S1** XRD patterns of the Ni_3_S_2_/NF, LES/NF, MES_T_/NF, MES_F_/NF and HES/NF.


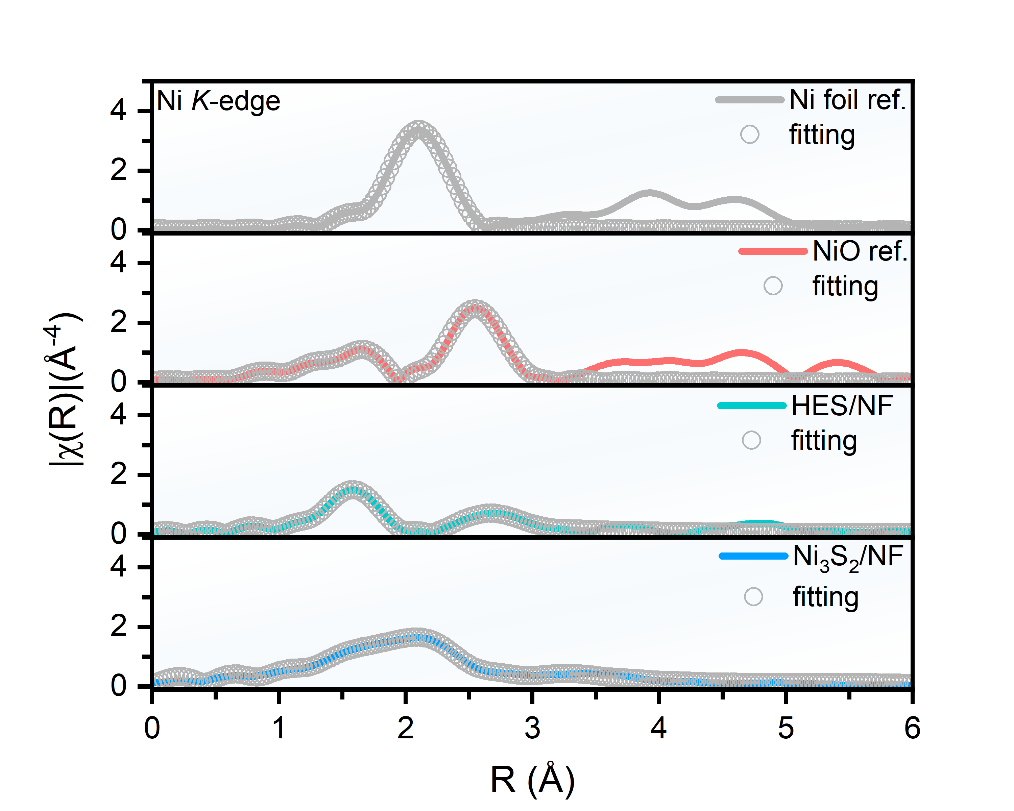


**Figure S2** Fitting results of Ni *K*-edge in *R*-space for Ni foil ref., NiO ref., HES/NF, and Ni_3_S_2_/NF.


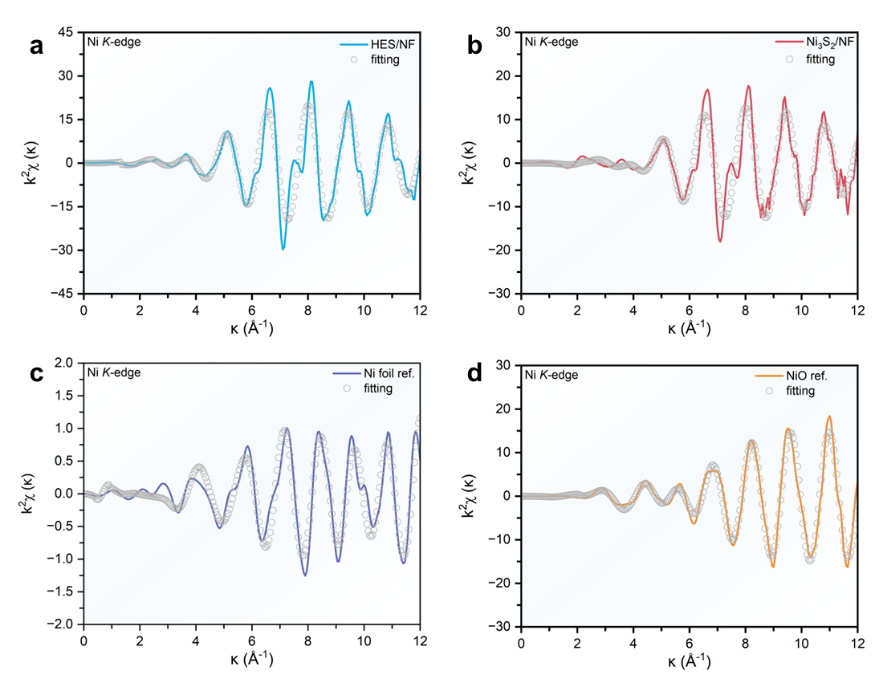


**Figure S3** Fitting results of Ni *K*-edge in *k*-space for Ni foil ref. (a), NiO ref. (b), HES/NF (c), and Ni_3_S_2_/NF (d).


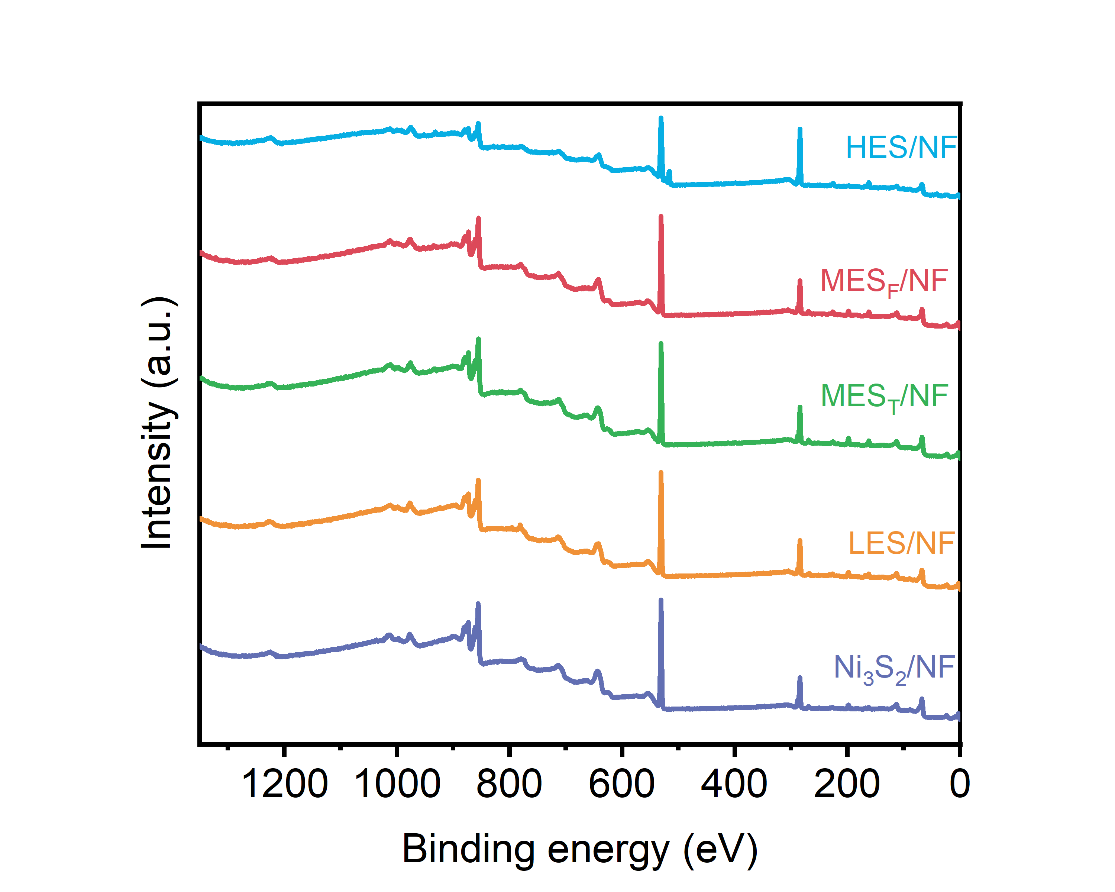


**Figure S4** The survey XPS spectrum of Ni_3_S_2_/NF, LES/NF, MES_T_/NF, MES_F_/NF and HES/NF.


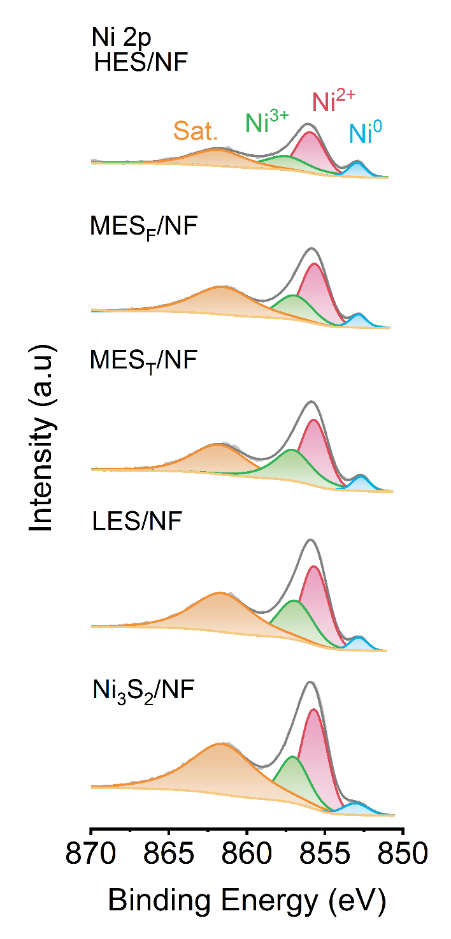


**Figure S5** The high-resolution Ni 2p of Ni_3_S_2_/NF, LES/NF, MES_T_/NF, MES_F_/NF and HES/NF.


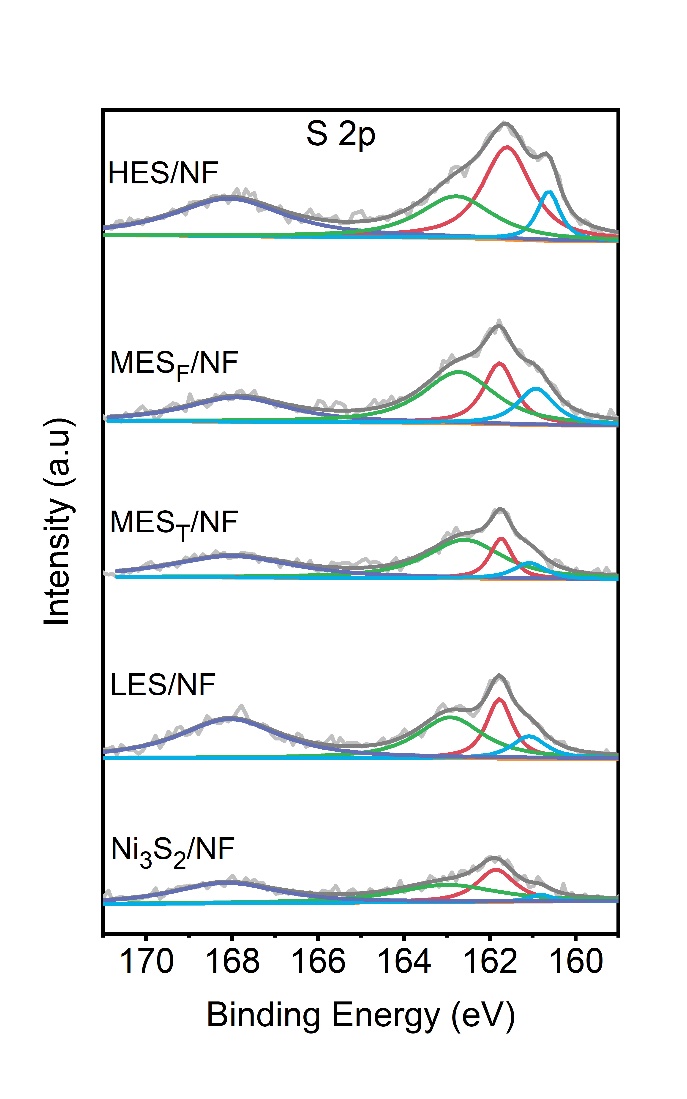


**Figure S6** The high-resolution S 2p XPS spectrum of Ni_3_S_2_/NF, LES/NF, MES_T_/NF, MES_F_/NF and HES/NF.


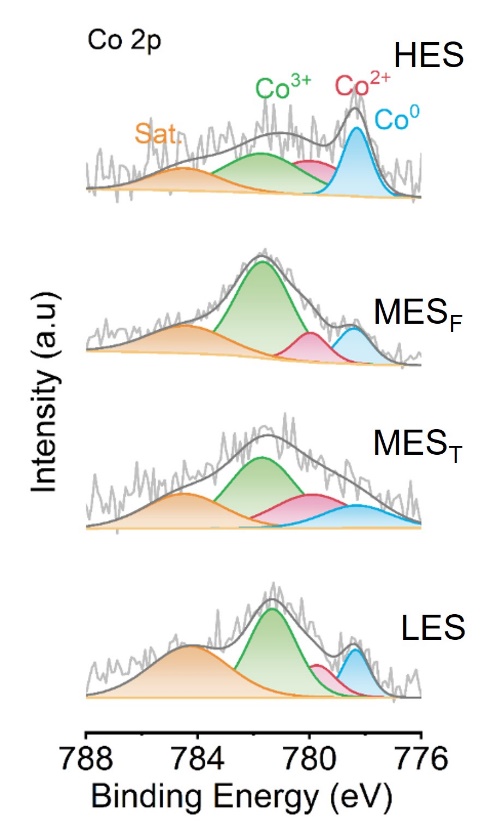


**Figure S7** The high-resolution Co 2p of LES/NF, MES_T_/NF, MES_F_/NF and HES/NF.

**
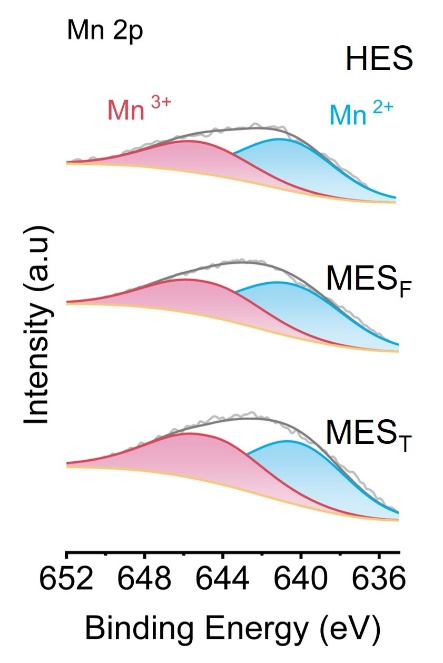
**

**Figure S8** The high-resolution Mn 2p of MES_T_/NF, MES_F_/NF and HES/NF.


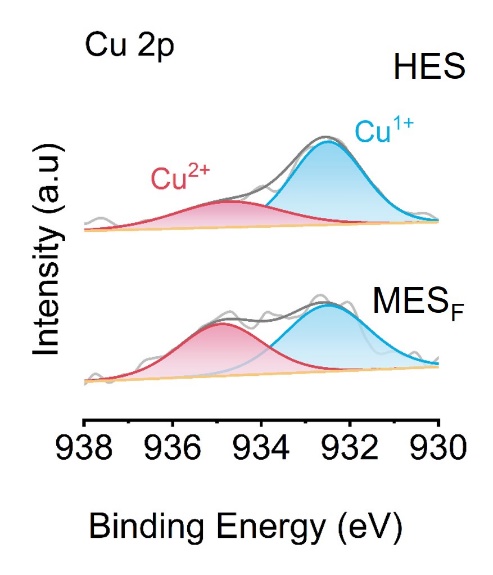


**Figure S9** The high-resolution Cu 2p of MES_F_/NF and HES/NF.


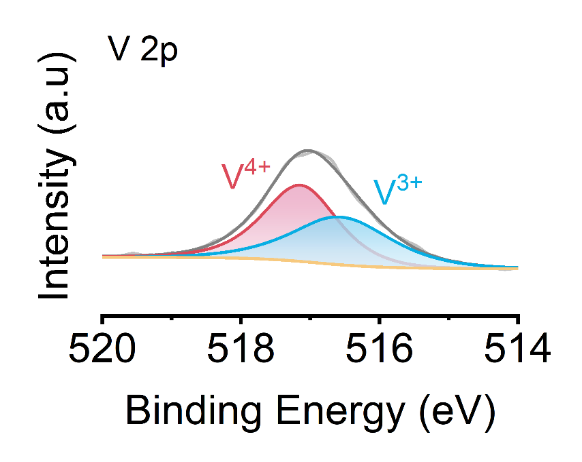


**Figure S10** The high-resolution V 2p of HES/NF.


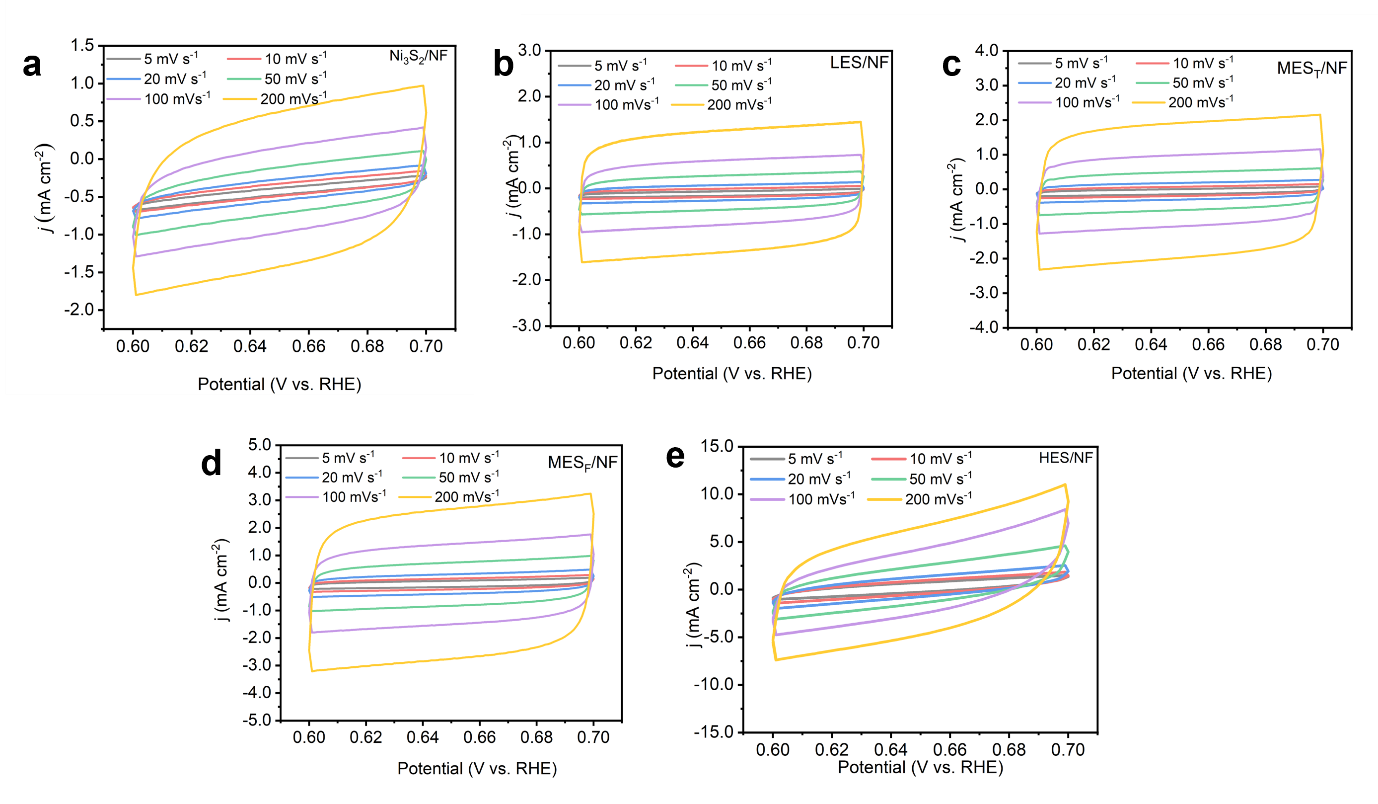


**Figure S11** The CV curves at different scan rates of Ni_3_S_2_/NF (a), LES/NF (b), MES_T_/NF (c), MES_F_/NF (d) and HES/NF (e).


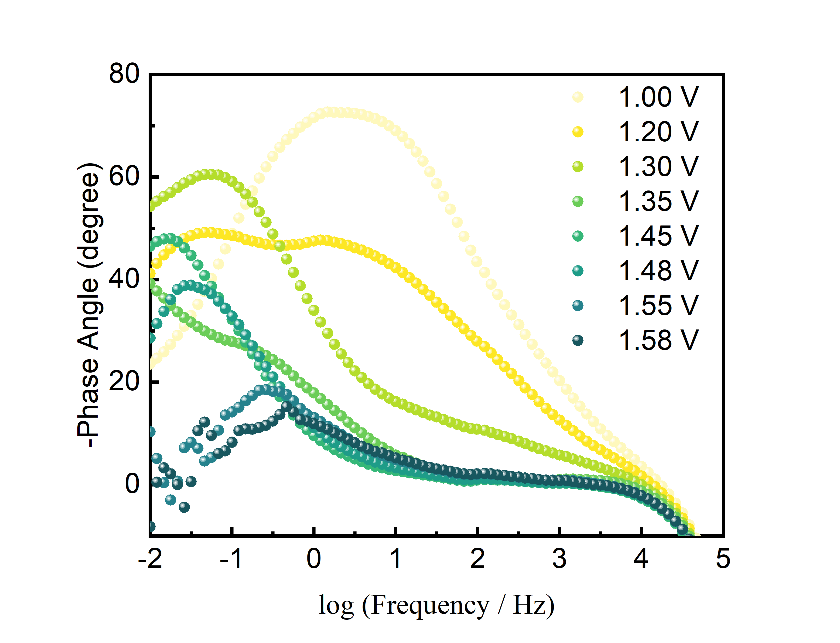


**Figure S12** *In-situ* EIS impedance testing diagram of the Ni_3_S_2_/NF.


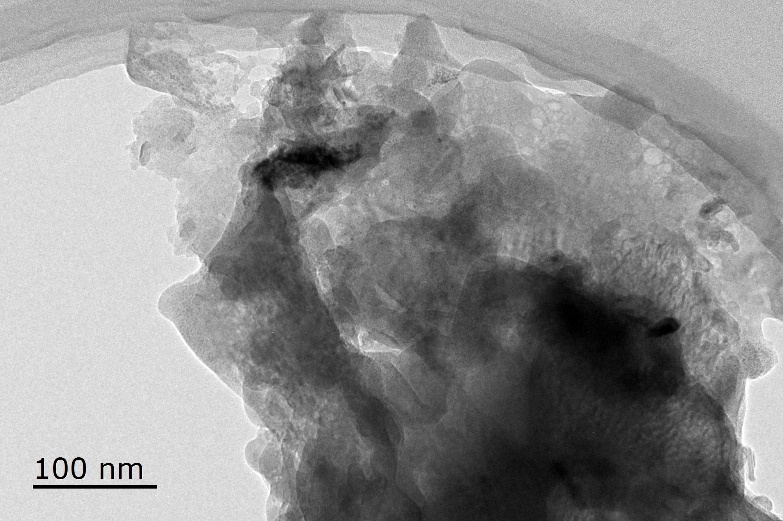


**Figure S13** The TEM image of the HES-MOOH/NF.


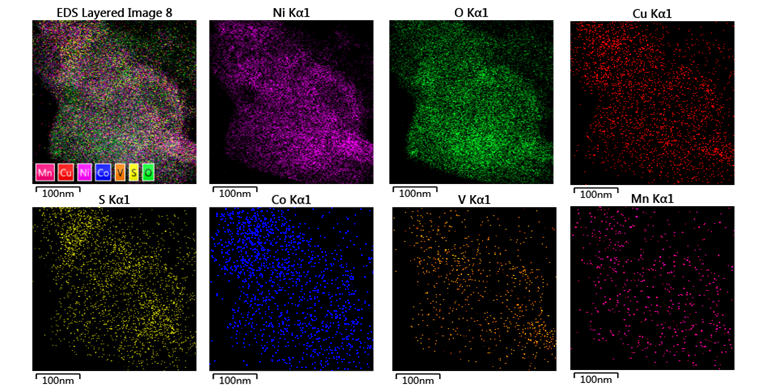


**Figure S14** EDX maps of the HES-MOOH/NF.


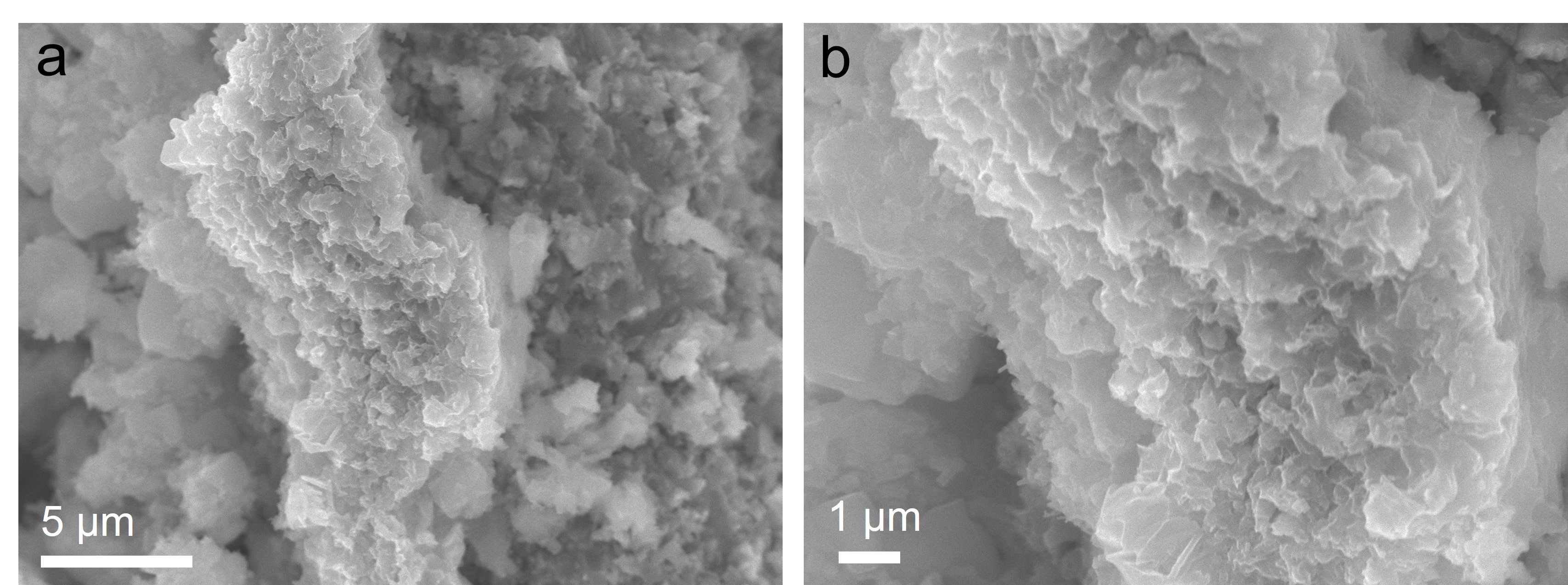


**Figure S15** The SEM image of the Ni_3_S_2_-NiOOH/NF.


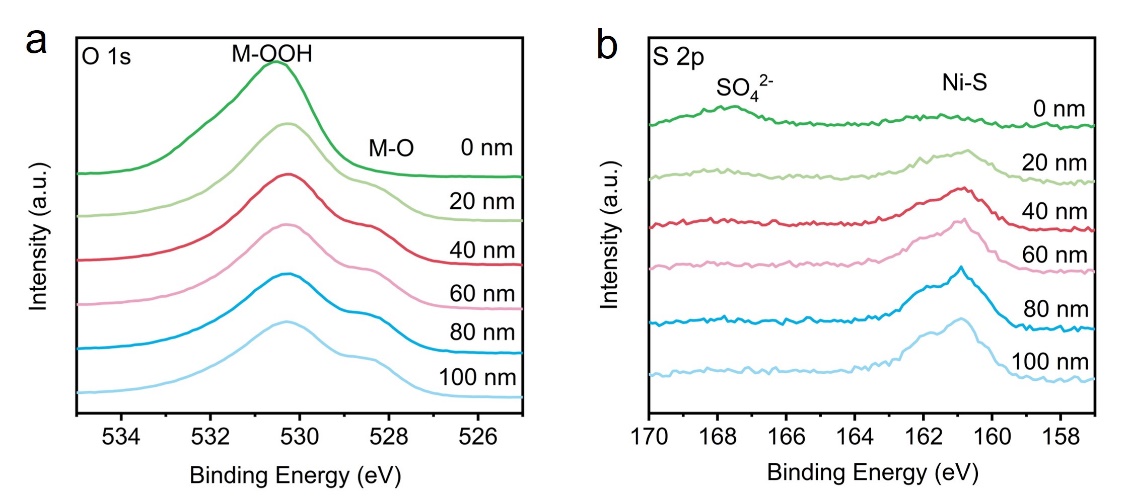


**Figure S16** High resolution XPS spectra of (a) O 1s and (b) S 2p of HES/NF at different etch depth.


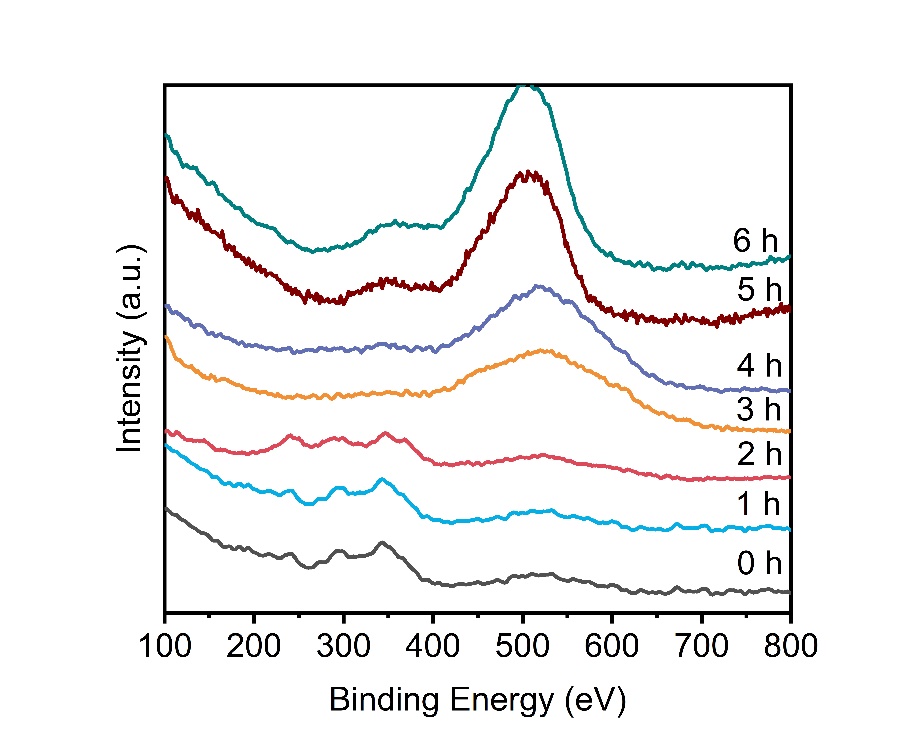


**Figure S17** The *ex-situ* Raman spectra of the Ni_3_S_2_/NF at different reaction times.


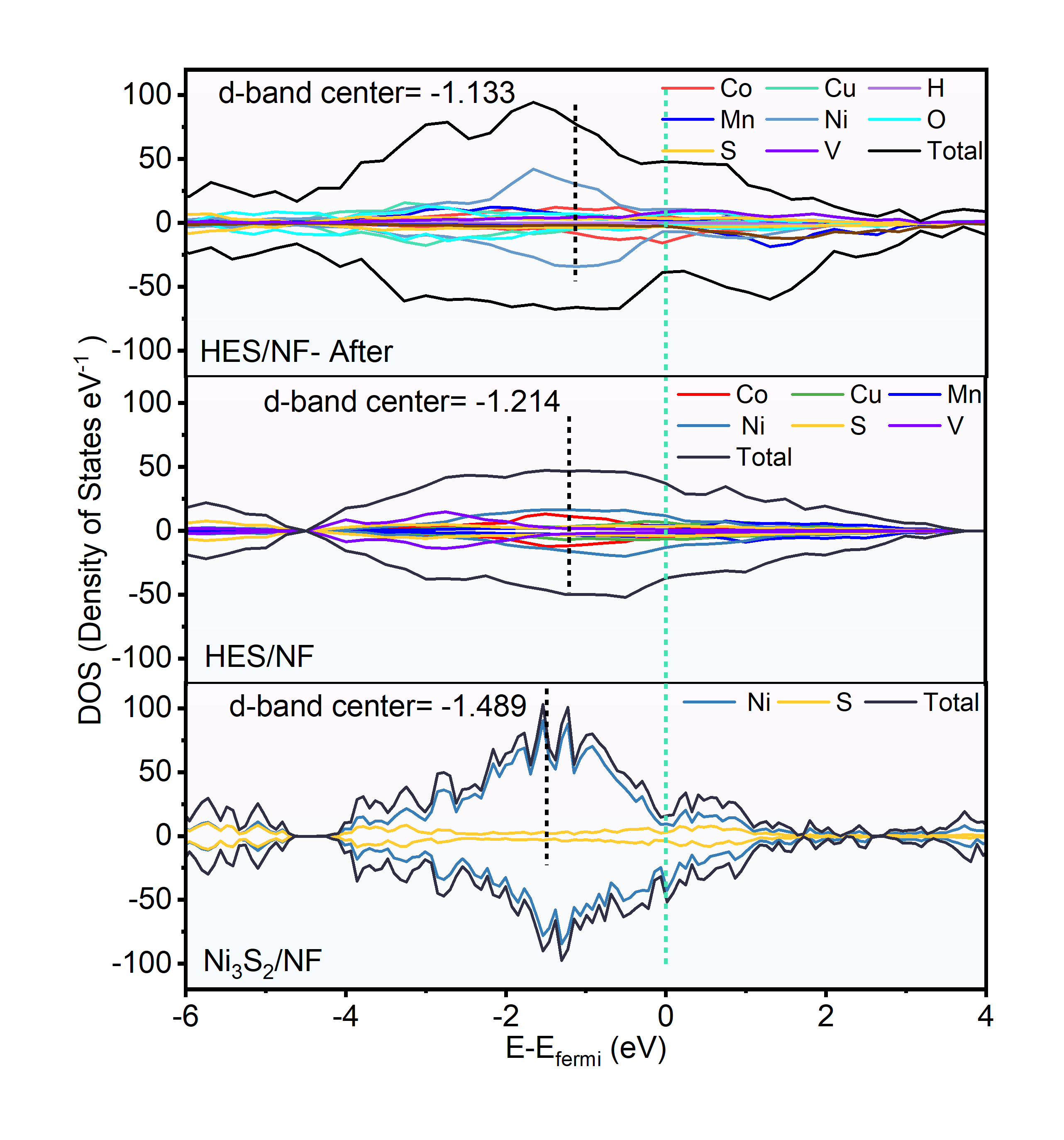


**Figure S18** The DOS of the Ni_3_S_2_/NF, HES/NF and HES-MOOH/NF.

**Table S1** The fitting parameters of Ni *K*-edge Fourier-filtered k^3^-weighted EXAFS for various samples.

| **Materials** | **Path** | **CN** | **R (Å)** | **Δ*Ε*_0_ (eV)** | **S_0_^2^** | **σ^2^ (Å^2^)** | ***R*-factor** |
| --- | --- | --- | --- | --- | --- | --- | --- |
| HES/NF | Ni-Ni | 4.8 | 3.01±0.01 | 7.9±1.0 | 0.90 | 0.0081±0.9 | 0.0102 |
|  | Ni-S | 4.5 | 2.04±0.02 | 5.0±0.8 | 0.90 | 0.0050±0.7 |  |
| Ni_3_S_2_/NF | Ni-Ni | 4.6 | 2.23±0.01 | -5.1±1.2 | 0.90 | 0.0074±0.5 | 0.0084 |
|  | Ni-S | 4.1 | 2.52±0.02 | -5.8±0.7 | 0.90 | 0.0093±0.6 |  |
| NiO ref. | Ni-Ni | 12 | 2.94±0.02 | -3.2±0.5 | 0.90 | 0.0066±0.8 | 0.052 |
|  | Ni-O | 6 | 2.06±0.01 | -1.0±0.3 | 0.90 | 0.0068±0.7 |  |
| Ni foil ref. | Ni-Ni | 12 | 2.49±0.02 | -5.4±0.8 | 0.90 | 0.0060±0.9 | 0.043 |

CN: coordination numbers, R: bond distance, *ΔΕ*_0_: the inner potential correction; σ_2_: Debye-Waller factors, R-factor: goodness of fit. S_0_^2^ was fixed as 0.90. Data ranges: 3 ≤ k ≤ 12 Å^-1^, 1.0 ≤ R ≤ 3.0 Å. For Ni foil ref. EXAFS fitting, the independent point is 11.5 and the number of variables is 4. For NiO ref. EXAFS fitting, the independent point is 11.4 and the number of variables is 7. For samples 1 EXAFS fitting, the independent point is 12.2 and the number of variables is 7. For samples 2 EXAFS fitting, the independent point is 11.2 and the number of variables is 4.

**Table S2** Atomic metal compositions in different sulfides and corresponding *Δ*S_mix_ values.

| **Catalysts** | **Metal**  **species** | | **Experimental At (%)** | **Experimental *Δ*S_mix_** | **Theoretical**  **At (%)** | | **Theoretical**  ***Δ*S_mix_** | |
| --- | --- | --- | --- | --- | --- | --- | --- | --- |
| Ni_3_S_2_/NF | Ni | 100.00 | | 0 | 100.00 | 0 | |  |
| LES/NF | Ni | 34.67 | | 0.69R | 50.00 | 0.69R | |  |
|  | Co | 34.68 | |  | 50.00 |  |  |  |
| MES_T_/NF | Ni | 18.65 | | 1.10R | 33.33 | 1.10R | |  |
|  | Co | 18.89 | |  | 33.33 |  |  |  |
|  | Mn | 18.47 | |  | 33.33 |  |  |  |
| MES_F_/NF | Ni | 24.98 | | 1.39R | 25.00 | 1.39R | |  |
|  | Co | 24.77 | |  | 25.00 |  |  |  |
|  | Mn | 25.73 | |  | 25.00 |  |  |  |
|  | Cu | 24.53 | |  | 25.00 |  |  |  |
| HES/NF | Ni | 22.25 | | 1.60R | 20.00 | 1.61R | |  |
|  | Co | 21.53 | |  | 20.00 |  |  |  |
|  | Mn | 18.48 | |  | 20.00 |  |  |  |
|  | Cu | 17.63 | |  | 20.00 |  |  |  |
|  | V | 20.09 | |  | 20.00 |  |  |  |

**Table S3** Comparison of OER performance among recently reported catalysts and our catalyst prepared in this work.

| **Samples** | **Overpotential (mV)** | **Overpotential @ current density** | **Tafel slopes** | ***C*_dl_ values** | **R_ct_** | **Ref.** |
| --- | --- | --- | --- | --- | --- | --- |
| **HES/NF** | **172 mV** | **100 mA cm^-2^** | **47.6 mV dec^−1^** | **13.2 mF cm^−2^** | **3.7 Ω** | **This work** |
| (NiCo)S_1.33_ | 302 mV | 10 mA cm^-2^ | 118 mV dec^−1^ | 19.9 mF cm^−2^ | 4.0 Ω | ^[1]^ |
| Ni(Fe)OOH–FeS*_x_* | 220 mV | 10 mA cm^-2^ | 55.0 mV dec^−1^ | 3.1 mF cm^−2^ | 2.2 Ω | ^[2]^ |
| HE-MHOF | 410 mV | 100 mA cm^−2^ | 57.0 mV dec^−1^ | — | — | ^[3]^ |
| HEA-HEO | 261 mV | 10 mA cm^−2^ | 42.2 mV dec^−1^ | 2.83 mF cm^−2^ | 1.81 Ω | ^[4]^ |
| K_0.8_Na_0.2_(MgMnFeCoNi)F_3_ | 314 mV | 10 mA cm^−2^ | 55.0 mV dec^−1^ | — | 5.0 Ω | ^[5]^ |
| Co–Cu–Fe–Mo (oxy) | 199 mV | 10 mA cm^−2^ | 48.8 mV dec^−1^ | 1.33 μF cm^−2^ | 4.0 Ω | ^[6]^ |
| *A*^5^Al_2_O_4_ | 400 mV | 10 mA cm^−2^ | — | — | — | ^[7]^ |
| HEO (CoFeNiCrMn) | 307 mV | 10 mA cm^−2^ | 34.7 mV dec^−1^ | 23.3 mF cm^−2^ | 26.9 Ω | ^[8]^ |
| FeCoNiCuMo-HEBs | 284 mV | 100 mA cm^−2^ | 54.0 mV dec^−1^ | 28.6 mF cm^−2^ | 3.3 Ω | ^[9]^ |
| HELHs | 259 mV | 100 mA cm^−2^ | 39.3 mV dec^−1^ | — | 8.0 Ω | ^[10]^ |
| Au_SA_-HE LDHs | 260 mV | 100 mA cm^−2^ | 27.5 mV dec^−1^ | — | 1.1 Ω | ^[11]^ |
| CNF@FeNiCo | 455.6 mV | 10 mA cm^−2^ | 71.5 mV dec^–1^ | — | — | ^[12]^ |
| Co/N-NiMo_3_S_4_ | 204 mV | 100 mA cm^-2^ | 41.0 mV dec^−1^ | — | — | ^[13]^ |
| Cu-ZnCoS/NF | 280 mV | 50 mA cm^−2^ | 111.9 mV dec^−1^ | 7.5 mF cm^2^ | — | ^[14]^ |
| (Ni,Fe)_x_S_y_ | 205 mV | 10 mA cm^−2^ | 38.37 mV dec^−1^ | — | 2.95 Ω | ^[15]^ |

**Table S4** Overpotentials comparisons of overall water splitting among recently reported catalysts towards and our catalyst in this work.

| **Samples** | **Overpotential (V) @100 mA cm^-2^** | **References** |
| --- | --- | --- |
| HES/NF | 350 | This work |
| MFN-0.5 | 480 | ^[16]^ |
| NiAu-MoS_2_ | 510 | ^[17]^ |
| N-Co_9_S_8_/Ni_3_S_2_ | 560 | ^[18]^ |
| FCNP@CQDs | 550 | ^[19]^ |
| Fe-CoP/Ni(OH)_2_ | 400 | ^[20]^ |
| NiMoN/NF-450 | 450 | ^[21]^ |
| CoTe_2_/CoP | 354 | ^[22]^ |
| Li, V co-doped Ni_3_S_2_ | 470 | ^[23]^ |
| ZnCoNiS | 620 | ^[24]^ |
| Ni_3_S_2_/FeNi_2_S_4_ | 630 | ^[25]^ |
| Fe-doped Ni_2_P | 420 | ^[26]^ |
| NiFeRh-LDH | 340 | ^[27]^ |
| Ni-Fe-Mo sulfide | 450 | ^[28]^ |

**References**

[1] Y. Hu, Y. Zheng, J. Jin, Y. Wang, Y. Peng, J. Yin, W. Shen, Y. Hou, L. Zhu, L. An, M. Lu, P. Xi, C.-H. Yan, *Nat. Commun.* **2023**, *14*, 1949.

[2] H. Yang, L. Gong, H. Wang, C. Dong, J. Wang, K. Qi, H. Liu, X. Guo, B. Y. Xia, *Nat. Commun.* **2020**, *11*, 5075.

[3] A. Roy, S. Kumar, A. Guilherme Buzanich, C. Prinz, E. Götz, A. Retzmann, T. Hickel, B. Bhattacharya, F. Emmerling, *Adv. Mater.*, **2024**, *36*, 2408114.

[4] J. Hu, T. Guo, X. Zhong, J. Li, Y. Mei, C. Zhang, Y. Feng, M. Sun, L. Meng, Z. Wang, B. Huang, L. Zhang, Z. Wang, *Adv. Mater.* **2024**, *36*, 2310918.

[5] T. Wang, H. Chen, Z. Yang, J. Liang, S. Dai, *J. Am. Chem. Soc*. **2020**, *142*, 4550-4554.

[6] L. Zhang, W. Cai, N. Bao, *Adv. Mater.* **2021**, *33*, 2100745.

[7] R. R. Katzbaer, F. M. dos Santos Vieira, I. Dabo, Z. Mao, R. E. Schaak, *J. Am. Chem. Soc*. **2023**, *145*, 6753-6761.

[8] J. Baek, M. D. Hossain, P. Mukherjee, J. Lee, K. T. Winther, J. Leem, Y. Jiang, W. C. Chueh, M. Bajdich, X. Zheng, *Nat. Commun.* **2023**, *14*, 5936.

[9] D. Liu, P. Guo, Q. Wang, X. Ding, Y. He, J. Zhou, D. Sun, H. Pan, R. Wu, *Adv. Mater.* **2025**, *37*, 2414067.

[10] Y. Ding, Z. Wang, Z. Liang, X. Sun, Z. Sun, Y. Zhao, J. Liu, C. Wang, Z. Zeng, L. Fu, M. Zeng, L. Tang, *Adv. Mater.* **2023**, 2302860.

[11] F. Wang, P. Zou, Y. Zhang, W. Pan, Y. Li, L. Liang, C. Chen, H. Liu, S. Zheng, *Nat. Commun.* **2023**, *14*, 6019.

[12] J.-H. Cha, S.-H. Cho, D.-H. Kim, D. Jeon, S. Park, J.-W. Jung, I.-D. Kim, S.-Y. Choi, *Adv. Mater.* **2023**, *35*, 2305222.

[13] S. Chandrasekaran, T. Ma, Z. Hu, Q. Liu, C. Zhan, Y. Li, C. Bowen, H. Lu, Y. Liu, *Appl. Catal. B Environ*. **2023**, *338*, 123007.

[14] J. Gautam, S.-Y. Lee, S.-J. Park, *Adv. Compos. Hybrid Mater.* **2024**, *7*, 155.

[15] M. Chen, W. Li, Y. Lu, P. Qi, H. Wu, K. Hao, Y. Tang, *Appl. Catal. B Environ*. **2024**, *358*, 124415.

[16] N. S. Gultom, C.-H. Li, D.-H. Kuo, M. Z. Silitonga, *Appl. Catal. B Environ*. **2024**, *353*, 124100.

[17] M. Kumar, T. C. Nagaiah, *J. Mater. Chem. A*. **2023**, *11*, 18336-18348.

[18] H. Xie, Y. Feng, X. He, Y. Zhu, Z. Li, H. Liu, S. Zeng, Q. Qian, G. Zhang, *Small* **2023**, *19*, 2207425.

[19] S. Lv, Y. Deng, Q. Liu, Z. Fu, X. Liu, M. Wang, Z. Xiao, B. Li, L. Wang, *Appl. Catal. B Environ*. **2023**, *326*, 122403.

[20] X. Yu, J. Zhao, M. Johnsson, *Adv. Funct. Mater.* **2021**, *31*, 2101578.

[21] Y. Wang, Y. Sun, F. Yan, C. Zhu, P. Gao, X. Zhang, Y. Chen, *J. Mater. Chem. A*. **2018**, *6*, 8479-8487.

[22] L. Yang, X. Cao, X. Wang, Q. Wang, L. Jiao, *Appl. Catal. B Environ*. **2023**, *329*, 122551.

[23] Q.-N. Ha, N. Susanto Gultom, C.-H. Yeh, D.-H. Kuo, *Chem. Eng. J.* **2023**, *472*, 144931.

[24] P. Li, L. Zhang, Y. Yao, T. Xie, W. Du, T. Zhao, J. Jiang, *Int. J. Hydrogen Energy* **2024**, *51*, 1521-1533.

[25] Y. Wu, Y. Li, M. Yuan, H. Hao, X. San, Z. Lv, L. Xu, B. Wei, *Chem. Eng. J.* **2022**, *427*, 131944.

[26] Y. Li, H. Zhang, M. Jiang, Q. Zhang, P. He, X. Sun, *Adv. Funct. Mater.* **2017**, *27*, 1702513.

[27] H. Sun, W. Zhang, J.-G. Li, Z. Li, X. Ao, K.-H. Xue, K. K. Ostrikov, J. Tang, C. Wang, *Appl. Catal. B Environ*. **2021**, *284*, 119740.

[28] Q. Li, W. Zhang, J. Shen, X. Zhang, Z. Liu, J. Liu, *J. Alloys Compd.* **2022**, *902*, 163670.
